# CircRNA inhibits DNA damage repair by interacting with host gene

**DOI:** 10.1186/s12943-020-01246-x

**Published:** 2020-08-24

**Authors:** Xiaolong Xu, Jingwei Zhang, Yihao Tian, Yang Gao, Xin Dong, Wenbo Chen, Xiaoning Yuan, Weinan Yin, Jinjing Xu, Ke Chen, Chunjiang He, Lei Wei

**Affiliations:** 1 School of Basic Medical Sciences, Wuhan University, Wuhan, 430071 Hubei China; 2 Hubei Key Laboratory of Tumor Biological Behaviors, Department of Breast and Thyroid Surgery, Hubei Cancer Clinical Study Center, Zhongnan Hospital, Wuhan University, Wuhan, 430071 Hubei China; 3 Department of Urology, Tongji Hospital, Tongji Medical College, Huazhong University of Science and Technology, Wuhan, 430030 China; 4 College of Biomedicine and Health, Huazhong Agricultural University, Wuhan, 430070 China; 5 Hubei Province Key Laboratory of Allergy and Immunology, Wuhan, 430071 Hubei China

**Keywords:** Breast cancer, circRNA, DNA damage repair, R-loop, Host gene

## Abstract

**Background:**

Deregulated circular RNAs (circRNAs) are associated with the development of cancer and therapy resistance. However, functional research of circRNAs mostly focus on potential miRNA or protein binding and more potential regulation of circRNA on host gene DNA in cancers are yet to be inspected.

**Method:**

We performed total RNA sequencing on clinical breast cancer samples and identified the expression patterns of circRNAs and corresponding host genes in patient blood, tumor and adjacent normal tissues. qPCR, northern blot and in situ hybridization were used to validate the dysregulation of circRNA circSMARCA5. A series of procedures including R-loop dot-blotting, DNA-RNA immunoprecipitation and mass spectrum, etc. were conducted to explore the regulation of circSMARCA5 on the transcription of exon 15 of SMARCA5. Moreover, immunofluorescence and in vivo experiments were executed to investigate the overexpression of circSMARCA5 with drug sensitivities.

**Results:**

We found that circRNAs has average higher expression over its host linear genes in peripheral blood. Compared to adjacent normal tissues, circSMARCA5 is decreased in breast cancer tissues, contrary to host gene SMARCA5. The enforced expression of circSMARCA5 induced drug sensitivity of breast cancer cell lines in vitro and in vivo. Furthermore, we demonstrated that circSMARCA5 can bind to its parent gene locus, forming an R-loop, which results in transcriptional pausing at exon 15 of SMARCA5. CircSMARCA5 expression resulted in the downregulation of SMARCA5 and the production of a truncated nonfunctional protein, and the overexpression of circSMARCA5 was sufficient to improve sensitivity to cytotoxic drugs.

**Conclusion:**

Our results revealed a new regulatory mechanism for circRNA on its host gene and provided evidence that circSMARCA5 may serve as a therapeutic target for drug-resistant breast cancer patients.

## Introduction

Circular RNAs (circRNAs) are novel RNAs that have been ubiquitously discovered in many species by high-throughput sequencing in recent years [1, 2]. CircRNAs are generated by the back-splicing of intronic, exonic or intergenic regions. circRNAs are resistant to RNase R, and the stability of their structures makes these molecules ideal candidates for disease [3]. Extensive studies have revealed that dysregulated circRNAs are involved in the development of various cancers. In gastric cancer, circRNAs, such as circPVT1, circLARP4, has_circ_0000096, and circ_100269, have been shown to play a role in promoting tumor growth, and their expression is correlated with high TNM stage and poor prognosis [4–7]. In colon and hepatic carcinoma, ciRS-7 promoted tumor development and progression by activating the EGFR and PI3K/Akt pathway [8, 9]. CircRNAs, such as circKIF4A, hsa_circ_0001944, hsa_circ_0001481 and circRNA_0025202, have been implicated in molecular typing, brain metastasis and drug resistance in breast cancer [10–12]. Although great progress has been made, the roles of circRNA and relevant molecular mechanisms remain largely unknown.

Previous studies have shown that circRNAs exert their functions in different ways. As noncoding RNAs, circRNAs regulate the expression of other genes by serving as sponges for microRNA and RNA-binding proteins [13, 14]. In addition, some circRNAs have been shown to be translated into functional proteins [15, 16]. In addition, circRNAs have also been shown to directly interact with the genomic DNA of the host gene in plant, which results in altered parent gene expression [17]. However, the interaction of circRNAs and host gene DNA were less studied in human cancers.

SMARCA5 is a member of the SWI/SNF complex with ATP-dependent chromatin remodeling activity [18–20]. In the process of DNA damage repair, SMARCA5 is involved in chromatin remodeling in DNA damage regions, providing a structural basis for the recruitment of DNA damage repair factors [21, 22]. In tumors, SMARCA5 is highly expressed in hepatic carcinoma and prostate cancer, and its expression level is inversely related to tumor radiosensitivity [23, 24].

In this study, we established circRNAs have average higher expression than their host genes in peripheral blood, comparing to tissues. Then we identified a circRNA derived from SMARCA5 (circSMARCA5) is significantly decreased in breast cancer cell lines and breast cancer samples. Different to previous works revealing circSMARCA5 can also function as a competing endogenous RNAs by binding with miRNA molecules [25–28], our mechanism exploration displayed circSMARCA5 is involved in regulating DNA repair capacity by binding exon DNA directly. And further functional investigation of this circRNA may contribute to the therapeutic implications for cytotoxic drug-resistant breast cancer patients.

## Results

### Identification of expression of circRNAs in breast cancer

**Fig. 1:**
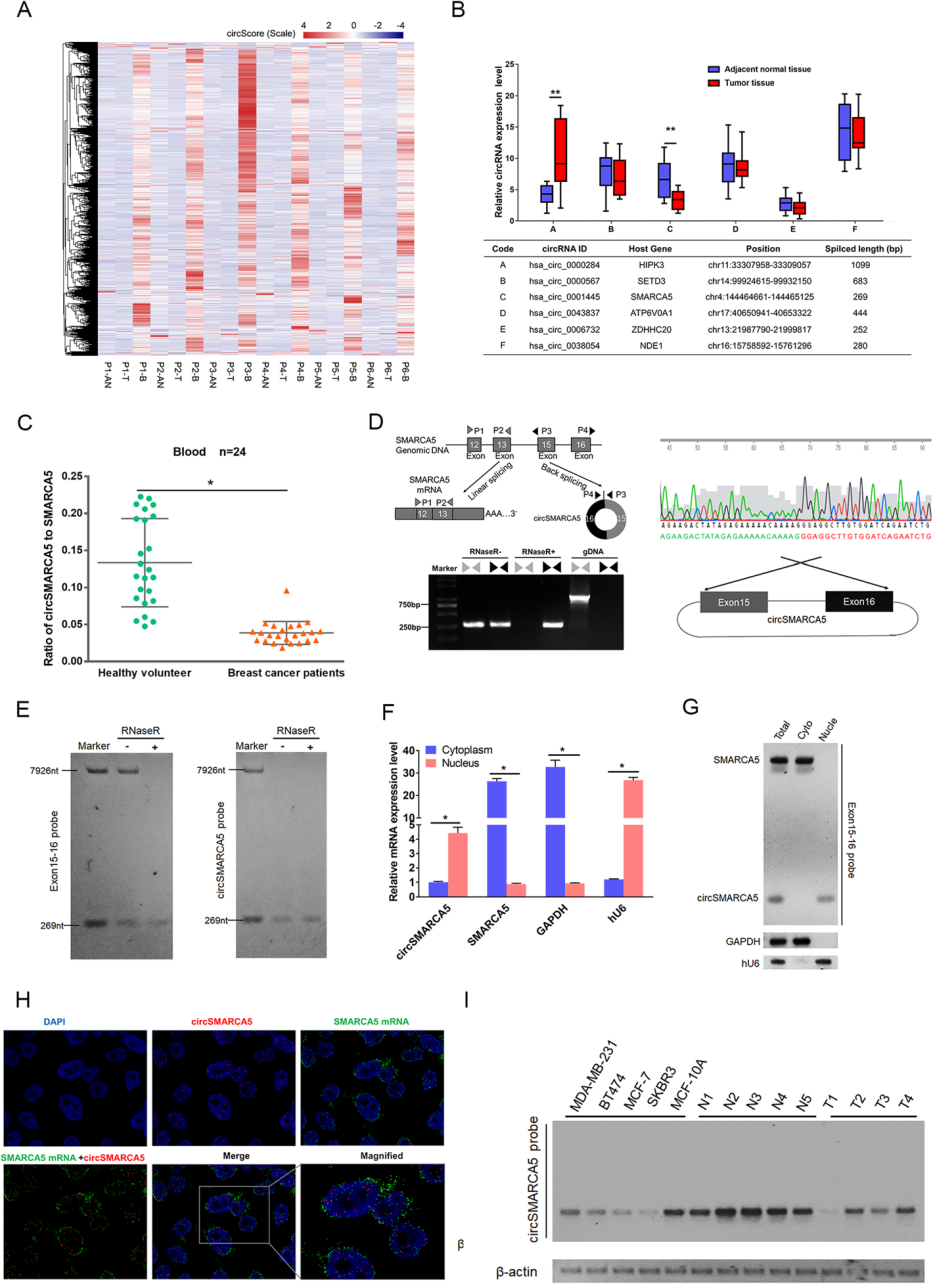
Identification of circRNAs in breast cancer. **a** Heatmap of CIRCscore (FBPcirc/FBPlinear) in tumor (T), adjacent normal tissue (AN) and blood sample (B) from six breast cancer patients. **b** Expression of six circRNAs with high CIRCscore were validated by RT-qPCR assay in breast tumor and adjacent normal tissue. ** represents *P* < 0.01. CircRNAs IDs are according to circBase through their genomic coordinates. **c** The ratio of circ-to-linear of circSMARCA5 in blood sample of breast cancer patients and health volunteers. Total RNA from blood sample of breast cancer patients and health volunteers was extracted and detected by RT-qPCR. The expression level was normalized with β-actin as reference. *: *P* < 0.05 was considered statistically significant. **d** Schematic illustration showing the genomic region of circSMARCA5 derived from exons 15 and 16 of the *SMARCA5* gene. Convergent (gray) and divergent (black) primers were designed to amplify the linear or back-splicing products (upper). Total RNA from MCF-7 cells with or without RNase R treatment was subjected to RT-PCR (lower) and further validated by Sanger sequencing (Right). **e** Northern blot using a junction-specific probe or an exon 15-16 probe showing the endogenous existence of circSMARCA5 and SMARCA5 mRNA from MCF-7 cells with or without RNase-R treatment (R+ or R-). The 7926 bp marker indicates the SMARCA5 full-length transcript transcribed *in vitro*. The 269 bp marker indicates exon 15 and exon 16 of SMARCA5 transcribed *in vitro*. **f** The nucleus and cytoplasm mRNA of MCF-7 were extracted, and SMARCA5 and circSMARCA5 expression levels were quantitated by RT-PCR. GAPDH and hU6 serve as internal references of the cytoplasm and nucleus, respectively. “**” indicates *P* < 0.01. **g** The nucleus and cytoplasm mRNA of MCF-7 were extracted, SMARCA5 and circSMARCA5 were examined by Northern blotting, and the SMARCA5 exon 15-16 probe was applied in this experiment. **h** Subcellular localization of circSMARCA5 and SMARCA5 in MCF-7 cells. The signals were examined by indirect RNA FISH and confocal microscopy. The nucleus was counterstained with DAPI. The circSMARCA5 probe was labled by biotin, while the SMARCA5 probe was labled by DIG. They were stained with red and green fluorescent secondary antibodies, respectively (I) The expression of circSMARCA5 detected by northern blot. MDA-MB-231, BT474, MCF-7, SKBR3 are breast cancer cell lines. MCF-10A are normal breast cell line. N1,N2,N3,N4,N5 are adjacent normal tissues. T1,T2,T3,T4 are breast cancer tissues. “**” indicates *P* < 0.01

We performed high throughput sequencing on tumor (T) and adjacent normal tissue (AN) and peripheral blood (B) of six breast cancer patients. Total RNA with rRNA-depleted library were constructed and then circRNAs expressed in those samples were identified. Compared to tumor and adjacent normal tissue, we observed average higher CIRCscore (expression of circRNA / linear host genes) in blood than both tumor and adjacent normal tissue. In all 8312 circRNAs which were expressed across all six patients, we observed average CIRCscores from 0.23 to 1.28 in blood, which is higher than tumor (0.05 to 0.11) and adjacent normal tissue (0.08 to 0.18) in six patients (Fig. 1a). This result indicated average higher expression of circRNAs than their host genes in peripheral blood, comparing to tissues, which might contribute to the exploration of diagnostic biomarker for breast cancer. We then selected six circRNAs with high CIRCscores (average 0.22 to 15.8 in 6 patients) and performed further experimental validation in 24 patients. Real-time PCR results established two circRNAs (circHIPK3 and circSMARCA5) were significantly differentially expressed between tumor and adjacent normal tissue (Fig. 1b and Figure S1A). Especially, circSMARCA5 was lower expressed in tumor samples and less studied in previous work. Furthermore, the ratio of circ-to-linear (expression of circRNA / linear host genes) of circSMARCA5 in blood sample of 24 health volunteers were significantly higher than those of 24 breast cancer patients (*P* < 0.05) (Fig. 1c and Figure S1B). We next examined the ratio of circ-to-linear of circSMARCA5 and clinical relevance in patients with breast cancer and observed significant difference in the distribution of the patients according to pathologic T (*P* = 0.038) (Table S1). Together, these results indicating the potential function and candidate biomarker attributes of circSMARCA5 in breast cancer.

To characterize and functionally investigate circSMARCA5, we firstly detected the expression of circSMARCA5 in cell lines. circSMARCA5 is derived from the back-splicing of exon 15 and exon 16 of SMARCA5 (Fig. 1d). As expected, endogenous circSMARCA5, but not pre-mRNA, was resistant to RNase R digestion (Fig. 1d). In addition, the existence of the 269 nt circSMARCA5 was further confirmed by Northern blot assay (Fig. 1e). Furthermore, we found that circSMARCA5 was mainly present in the nucleus, whereas its parent mRNA was present exclusively in the cytoplasm, as evidenced by qPCR, Northern blotting and RNA in situ hybridization (Fig. 1f-h and Figure S2). Next, we examined the expression of circSMARCA5 in various breast cancer cell lines (MCF7, SKBR3, BT474, MDA-MB-231) and immortalized but nontransformed breast epithelial cells (MCF-10A) as well as in adjacent normal tissues and breast cancer tissues. Northern blot results revealed that the expression levels of circSMARCA5 in MCF-10A and normal adjacent tissues are higher than breast cancer cell lines and cancer tissues (Fig. 1i). These results indicated that circSMARCA5 is downregulated in breast cancer tissues and cells.

### circSMARCA5 decreases the expression of SMARCA5 in cancer cells

**Fig. 2:**
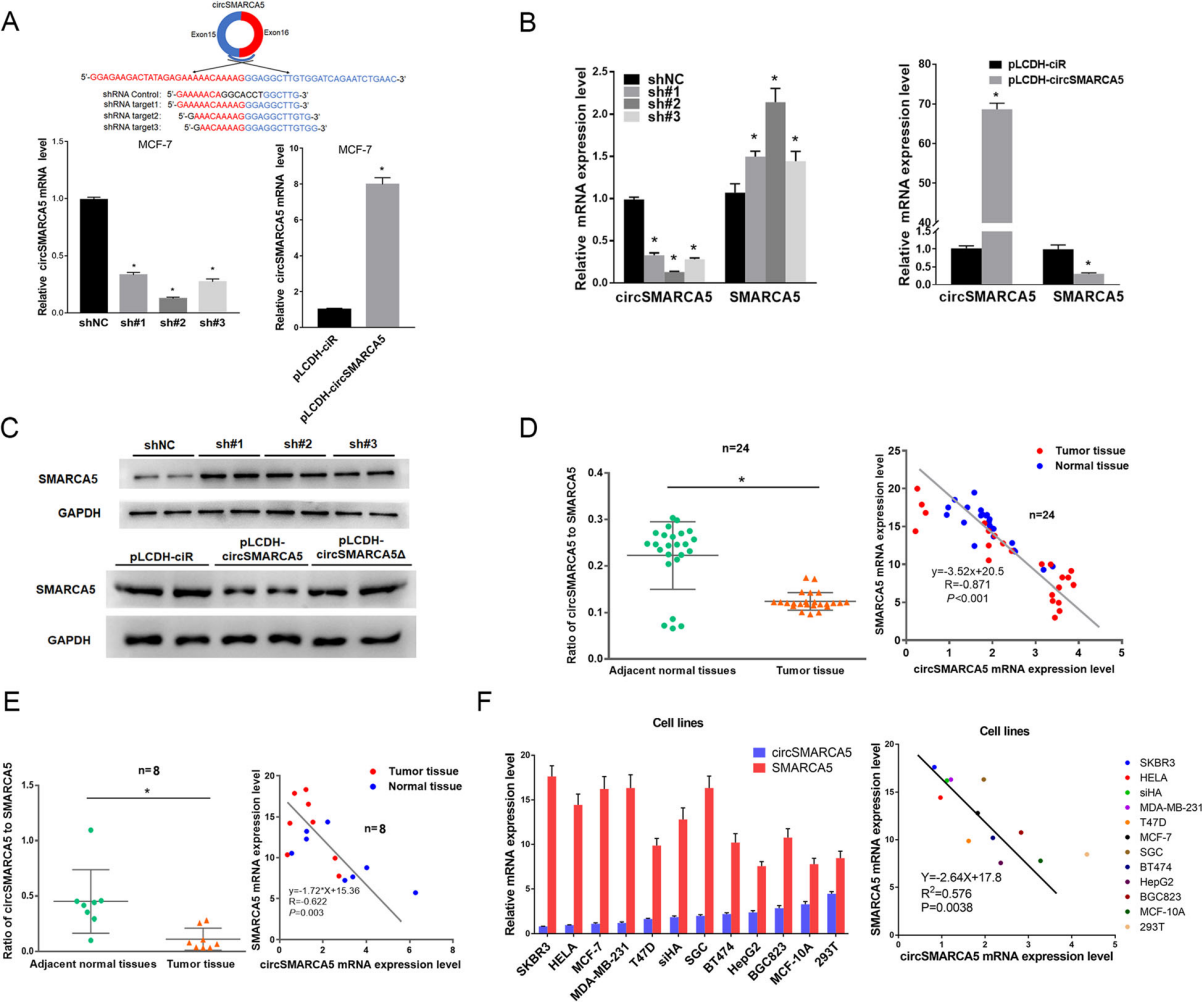
circSMARCA5 decreases the expression of SMARCA5 in cells. **a** Generation of circSMARCA5-knockdown and circSMARCA5-overexpressing cells. MCF-7 cells were infected with lentiviruses expressing shRNA against circSMARCA5 (sh-circSMARCA5; three different oligonucleotides) or circSMARCA5 (pLCDH-circSMARCA5). RT-qPCR was performed to evaluate the expression of circSMARCA5. GAPDH was used as an internal control. **b** RT-qPCR showing the levels of circSMARCA5 and SMARCA5 in MCF-7 cells stably expressing sh-NC, sh-circSMARCA5, pLCDH-ciR (control), or pLCDH-circSMARCA5. **c** Western blot showing the levels of SMARCA5 in MCF-7 cells stably expressing sh-NC, sh-circSMARCA5, pLCDH-ciR (control), pLCDH-circSMARCA5, pLCDH-circSMARCA5Δ (without splicing-inducing sequence). GAPDH was used as an internal control. (D-F) The ratio of circ-to-linear of circSMARCA5 in tumor tissue were significantly lower than normal tissue in breast cancer samples (**d**) and RCC samples (**e**) (*P* < 0.05). A negative correlation between circSMARCA5 and SMARCA5 expression was observed in breast cancer samples (**d**), RCC samples (**e**), and various cell lines (**f**)

To clarify the mechanisms of circSMARCA5, we investigated its effects on the expression of its parent gene *SMARCA5*. The expression levels of circSMARCA5 and SMARCA5 were detected by the primers of junction sequence and 22–23 exons sequence, respectively. Knockdown of circSMARCA5 increased both mRNA and protein levels of SMARCA5, while conversely, circSMARCA5 overexpression decreased SMARCA5 levels (Fig. 2a-c and Figure S3). Consistently, the protein of SMARCA5 was high expressed in breast tumor samples as compared with the corresponding controls (Figure S4). Moreover, the ratio of circ-to-linear of circSMARCA5 was significantly lower in breast and renal tumor tissue than the corresponding adjacent tissue specimens (Fig. 2d-e and Figure S1C). Besides, a significant negative correlation was also found between circSMARCA5 and SMARCA5 expression in various cell lines and primary cancer tissues (Fig. 2d-f and Figure S5), which corroborates our observation that circSMARCA5 decreased the expression of SMARCA5 in cancer cells.

### cirSMARCA5 terminates the transcription of SMARCA5 at exon 15

**Fig. 3:**
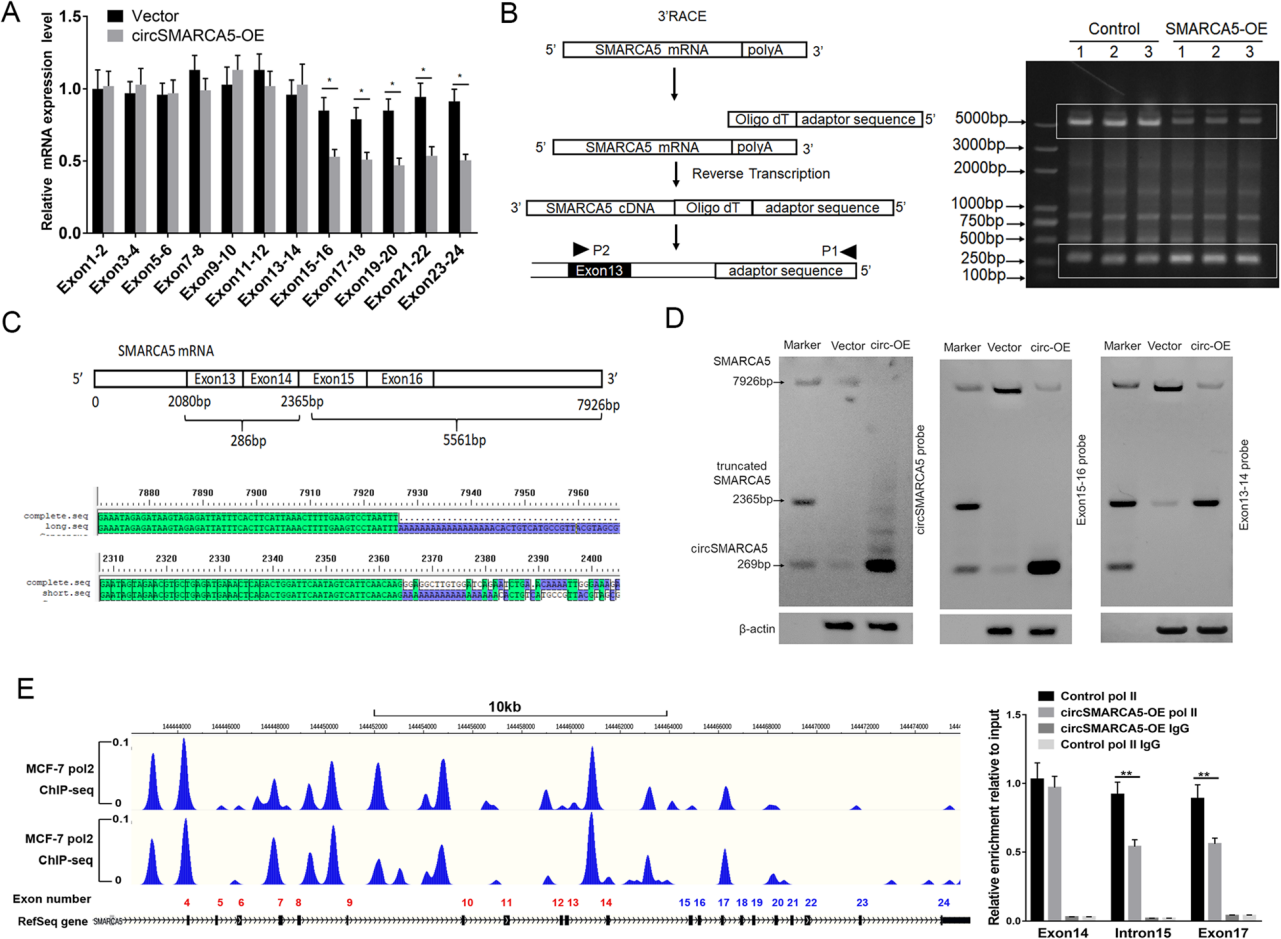
cirSMARCA5 terminates the transcription of SMARCA5 at exon 15. **a** RT-qPCR analysis of the expression of SMARCA5 in MCF-7 cells using a series of paired primers. “*” indicates *P* < 0.05. **b** Rapid amplification of cDNA ends (RACE) PCR analysis of SMARCAC5 transcripts. The PCR products were readily identified by agarose gel electrophoresis. Each set of samples was repeated three times. **c** Sanger sequencing of two transcripts of SMARCAC5 that are regulated by circSMARCA5 overexpression. **d** Northern blotting using the junction-specific probes for exons 13-14 and 15-16 to show the expression levels of the transcripts of SMARCAC5 mRNA from MCF-7 cells stably expressing control vector or pLCDH-circSMARCA5 (circ-OE). **e** CircSMARCA5 prevents transcription from exon 15 of SMARCA5. ChIP-seq analysis showing that the binding of pol II to exons 1-14 of SMARCA5 was higher than that to exons 15-24. ChIP-qPCR showed that the ectopic expression of circSMARCA5 decreased the binding of Pol II to exons 15-24 of SMARCA5

**Fig. 4:**
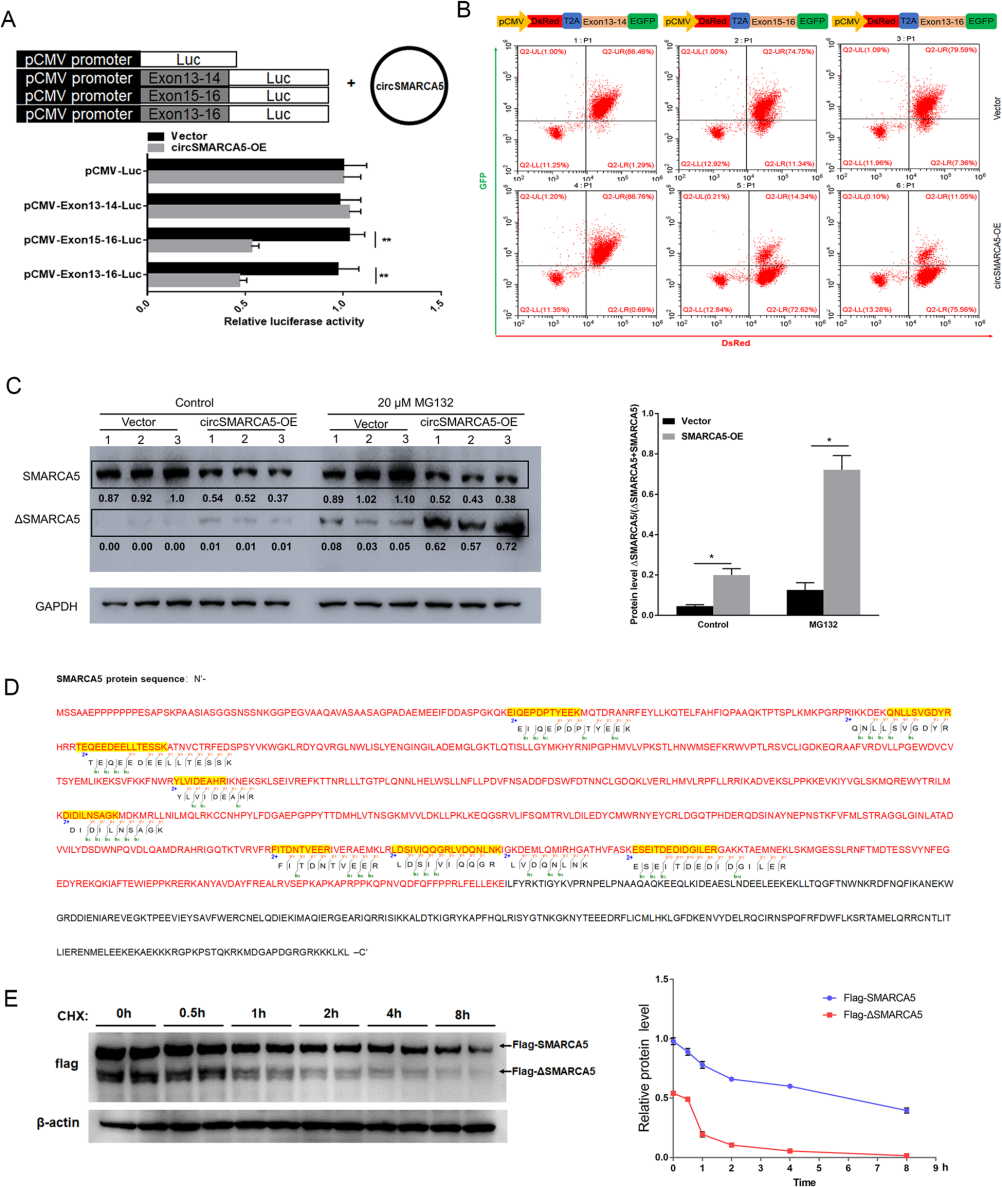
cirSMARCA5 blocks the transcription of SMARCA5 and promotes the generation of a truncated SMARCA5 protein (ΔSMARCA5). **a** Schematics of luciferase reporter constructs containing the SMARCA5 exon sequence as indicated (upper). The SMARCA5 exon 15-16 sequence plays an important negative role in mediating the effect of circSMARCA5 overexpression on luciferase activity (lower). **b** Schematics of fluorescence reporter constructs containing the SMARCA5 exon sequence as indicated (upper). MCF-7 cells were transiently transfected with these fluorescence reporters along with or without circSMARCA5 co-overexpression. After transfection for 48 hours, the reporter transcription activities were measured by flow cytometry assay. **c** circSMARCA5 overexpression downregulated the protein levels of SMARCA5 while upregulating truncated SMARCA5 (ΔSMARCA5) protein levels. MCF-7 cells stably overexpressing circSMARCA5 or control cells were treated with DMSO or MG132. Western blot analysis was performed using an antibody targeting the N-terminus of SMARCA5 to evaluate the expression of SMARCA5 and ΔSMARCA5. GAPDH was used as an internal control. **d** The ΔSMARCA5 protein was identified by mass spectrometry, and detected SMARCA5 peptides were showed in the map. The red-labeled portion is the amino acid sequence of the translated defective transcript. **e** MCF-7 cells expressing Flag-SMARCA5 and Flag-ΔSMARCA5 were treated with cycloheximide (CHX, 50 μg/ml). The cell lysates were subsequently harvested at sequential time points (0, 0.5, 1, 2, 4 or 8 h) after treatment, and then the cell lysates were immunoblotted with anti-Flag or anti-Actin antibody

We further investigated the mechanism of circSMARCA5 in regulating the expression of SMARCA5. Interestingly, we found that the overexpression of circSMARCA5 indeed decreased the expression of SMARCA5 exons 15–24 but had minimal effects on the expression of exons 1–14 (Fig. 3a). Next, we designed a primer location in exon 13 for the amplification of 3′ cDNA ends by rapid amplification of cDNA ends (RACE) PCR (Fig. 3b, left). As shown in Fig. 3b, SMARCA5 can give rise to multiple isoforms. Importantly, we found a decrease in a band of ~ 5000 bp upon circSMARCA5 overexpression, while an ~ 250 bp band displayed the opposite phenomenon (Fig. 3b, right). Sanger sequencing showed that the ~ 5000 bp band and the ~ 250 bp band are derived from full-length and truncated mRNA (exons 1 to 14), respectively, of the SMARCA5 gene (Fig. 3c). Consistent with the RACE results, Northern blot assay further demonstrated that ectopic circSMARCA5 expression decreased SMARCA5 levels and promoted truncated mRNA levels (Fig. 3d). The observations gathered thus far have led us to hypothesize that circSMARCA5 prevents transcription from exon 15 of SMARCA5. Indeed, ChIP analysis indicated that the binding of pol II to exons 1–14 of SMARCA5 was higher than that to exons 15–24 (Fig. 3e, left), and the ectopic expression of circSMARCA5 decreased the binding of Pol II to exons 15–24 of SMARCA5 (Fig. 3e, right). To further address whether circSMARCA5 could terminate the transcriptional elongation of SMARCA5, we cloned a series of exons of SMARCA5 in a luciferase plasmid reporter (Fig. 4a, upper). The transient transfection of these luciferase reporters containing the 15–16 exon sequence revealed that luciferase activity was significantly decreased when circSMARCA5 was overexpressed (Fig. 4a, lower). To further confirm the effect of circSMARCA5 on the transcriptional elongation of SMARCA5, we inserted exons of SMARCA5 between DsRED and EGFP as indicated (Fig. 4b, upper). The EGFP level was significantly decreased by circSMARCA5 when exons 15–16 were present (Fig. 4b, lower). We further investigated the role of circSMARCA5 in the regulation of SMARCA5 at the protein level. As expected, circSMARCA5 overexpression downregulated the protein levels of SMARCA5 and upregulated truncated SMARCA5 (ΔSMARCA5) protein levels (Fig. 4c and Figure S6), which was confirmed by mass spectrometry (Fig. 4d). Moreover, we found that ΔSMARCA5 is more susceptible to proteolysis by the proteasome than SMARCA5 (Fig. 4e). Together, these results show the role of circSMARCA5 in the termination of transcriptional elongation at exon 15 of SMARCA5.

### circSMARCA5 can form R-loops with its parent gene

**Fig. 5:**
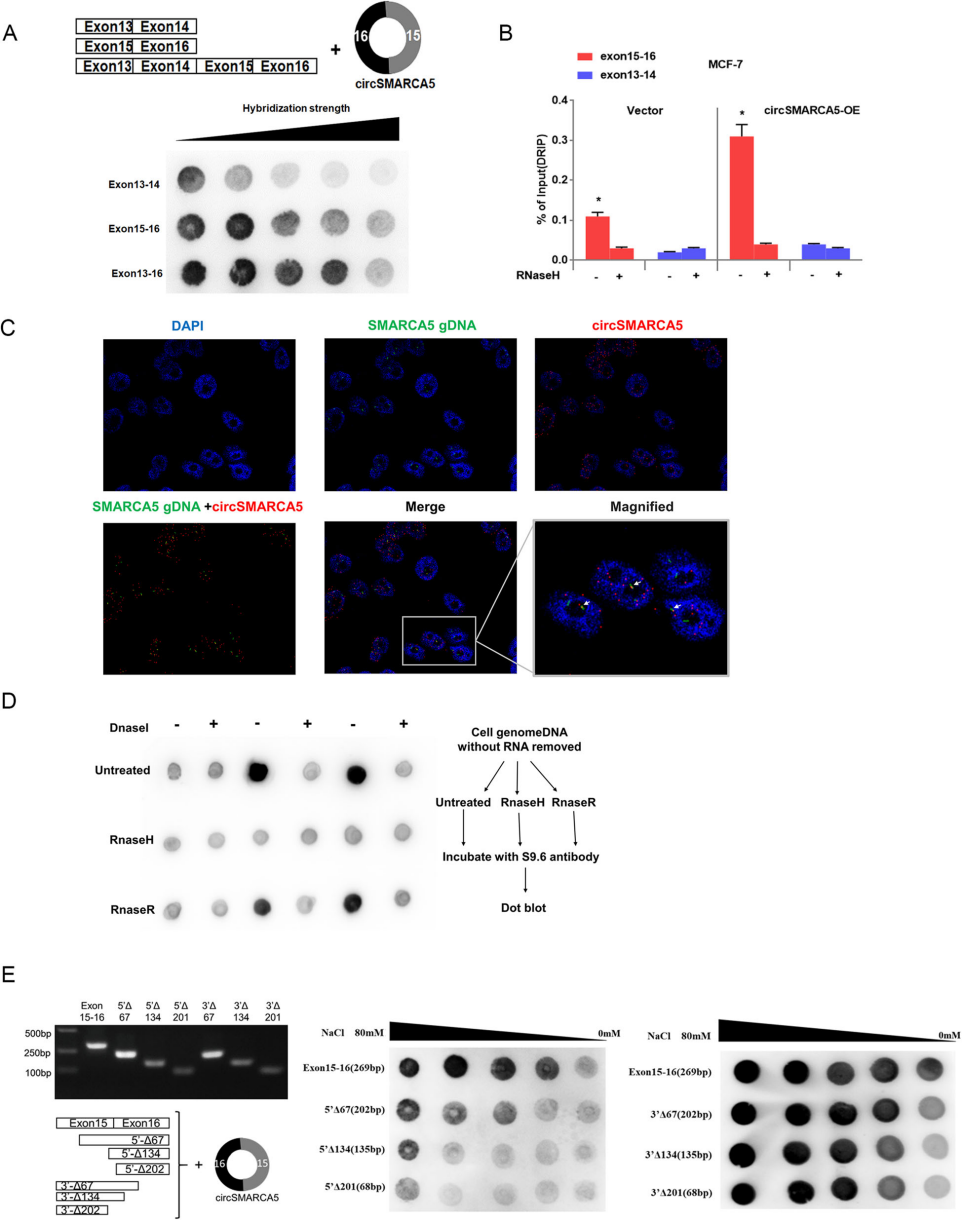
circSMARCA5 interacts with its site of transcription. **a** circSMARCA5 interacts with the 15-16 exon sequence of the SMARCA5 locus. A series of exon DNA fragments were hybridized with circSMARCA *in vitro*. The DNA-RNA hybridization strength was quantified by dot-blot with R-loop-specific S9.6 antibody. Hybridization stringency was altered by decreasing ionic strength (80-0.1 mM NaCl). **b** DRIP-qPCR analysis of the 15-16 exon sequence of SMARCA5 to detect the association of circSMARCA5 in MCF-7 cells. RNase H-treated and/or DRIP-qPCR analysis of the 15-16 exon sequence as a control. **c** CircSMARCA5 partially localized at its site of transcription. Double FISH of circSMARCA5 (red) and its parent DNA region (green). The nucleus was stained by DAPI. **d** Dot-blot of R-loops in MCF-7 cell genomic DNA preparations treated with DNase I, RNase H, or RNase R. The DNA-RNA hybrids in genome DNA were analyzed by S9.6 antibody. **e** Mapping of the R-loop formation region of circSMARCA5. A series of exon 15-16 deletion mutants were hybridized with circSMARCA5 for the dot-blotting assay. The DNA-RNA hybridization intensity was analyzed by dot-blot with an S9.6 antibody targeting the DNA-RNA hybrid strand. Hybridization stringency was altered by decreasing ionic strength (80-0.1 mM NaCl)

**Fig. 6:**
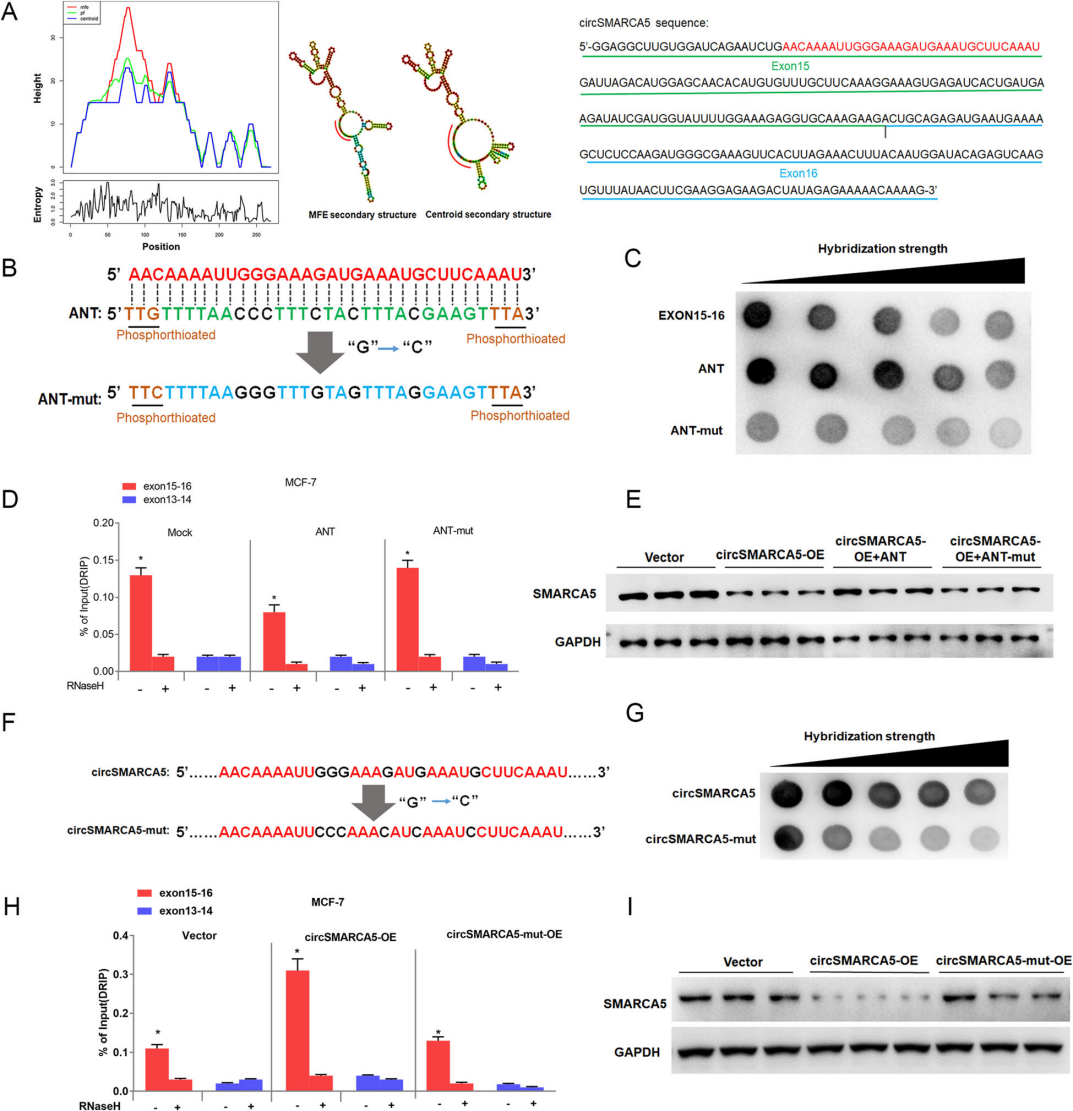
circSMARCA5 can form an R-loop with its parent gene. **a** Secondary structure prediction for circSMARCA5 using the Mfold program. The sequence KEY shared by the minimum free energy structure and the thermodynamic ensemble structure is marked by red. **b** The thiophosphorus nucleic acid analog ANT complementary to KEY and its mutant ANT-mut were synthesized *in vitro*. *c* Dot-blot verifying the interaction between circSMARCA and ANT or ANT-mut. **d** DRIP-qPCR analysis on 15-16 exon or 13-14 exon sequences of SMARCA5 to detect the association of circSMARCA5 in MCF-7 cells overexpressing ANT or ANT-mut, RNase H-treated and/or DRIP-qPCR analysis of the 15-16 exon sequence as a control. “*” indicates *P* < 0.05. **e** Western blot analysis shows that transfection of ANT into circSMARCA5-overexpressing cells can restore SMARCA5 protein levels but ANT-mut cannot. (**f**, **g**) Dot-blot analysis quantifying R-loop strength between the SMARCA5 locus and circSMARCA5 or circSMARCA5-mut (guanine converted to cytosine of the KEY sequence). **h** DRIP-qPCR in MCF-7 cells transfected with circSMARCA5 or circSMARCA5-mut. RNase H-treated genomic DNA and qPCR of exon13-14 were treated as controls. “*” indicates *P* < 0.05. **i** Western blot analysis shows that overexpression of circSMARCA5 to MCF-7 cells can decrease SMARCA5 protein levels but circSMARCA5-mut cannot

To further dissect the mechanism of SMARCA5 transcriptional termination mediated by circSMARCA5, we investigated whether circSMARCA5 can bind genomic SMARCA5 DNA to form an R-loop. Dot-blotting with R-loop-specific S9.6 antibody supported our hypothesis that circSMARCA5 can bind exons 15–16 of SMARCA5 genomic DNA (Fig. 5a). Additionally, we performed DNA-RNA immunoprecipitation (DRIP) qPCR and confirmed the interaction between circSMARCA5 and exons 15–16; pretreatment with RNase H ablated this interaction, confirming that the interaction is R-loop-specific (Fig. 5 b and Figure S7). The interaction of circSMARCA5 with the DNA of SMARCA5 was directly verified by fluorescence in situ hybridization (Fig. 5c). Consistent with previous findings [17], dot-blotting of the genome without RNA digest revealed that the binding of circRNA to genomic DNA may be widely present in cancer cells (Fig. 5d). We next determined the specific sequence of exons 15–16 required for R-loop formation. A series of fragments from exons 15–16 were hybridized with circSMARCA5 for the dot-blotting assay. As shown in Fig. 5e, the 67 bp fragment of the 5′ end of exon 15 plays important role in interacting with circSMARCA5. Moreover, the secondary structure of circSMARCA5 was determined by the software MFOLD [29], which revealed the sequence 5′-AACAAAAUUGGGAAAGAUGAAAUGCUUCAAAU-3′ from the 5′ end of exon 15 located in the loop region of circSMARCA5 (Fig. 6a). We thus hypothesized that this sequence might play a key role in mediating the circSMARCA5-DNA interaction. To this end, we synthesized the wild-type and mutant phosphorylated DNA fragments, ANT and ANT-mut, respectively, corresponding to this sequence (Fig. 6b). Dot-blotting demonstrated that wild-type oligonucleotides (ANT) can bind to circSMARCA5, but mutant oligonucleotides (ANT-mut) cannot bind to circSMARCA5 (Fig. 6c). As expected, DRIP-qPCR showed that ANT inhibited circSMARCA5 binding to the DNA at exons 15–16, whereas ANT-mut had no effect on this interaction (Fig. 6d). Furthermore, the transfection of ANT prevented the decrease in SMARCA5 protein levels in MCF-7 cells stably expressing circSMARCA5, whereas ANT-mut had no effect on SMARCA5 protein levels (Fig. 6e). Importantly, the mutation of the key sequence in circSMARCA5 impaired the interaction with its parent gene, which was confirmed by dot-blotting and DRIP-qPCR assays (Fig. 6f-h and Figure S8). Unlike circSMARCA5, circSMARCA5-mut had little effect on SMARCA5 protein levels (Fig. 6i). These results suggested that circSMARCA5 formed R-loops with its parent gene to inhibit the expression of SMARCA5 in cancer cells.

### circSMARCA5 inhibits DNA damage repair function

**Fig. 7:**
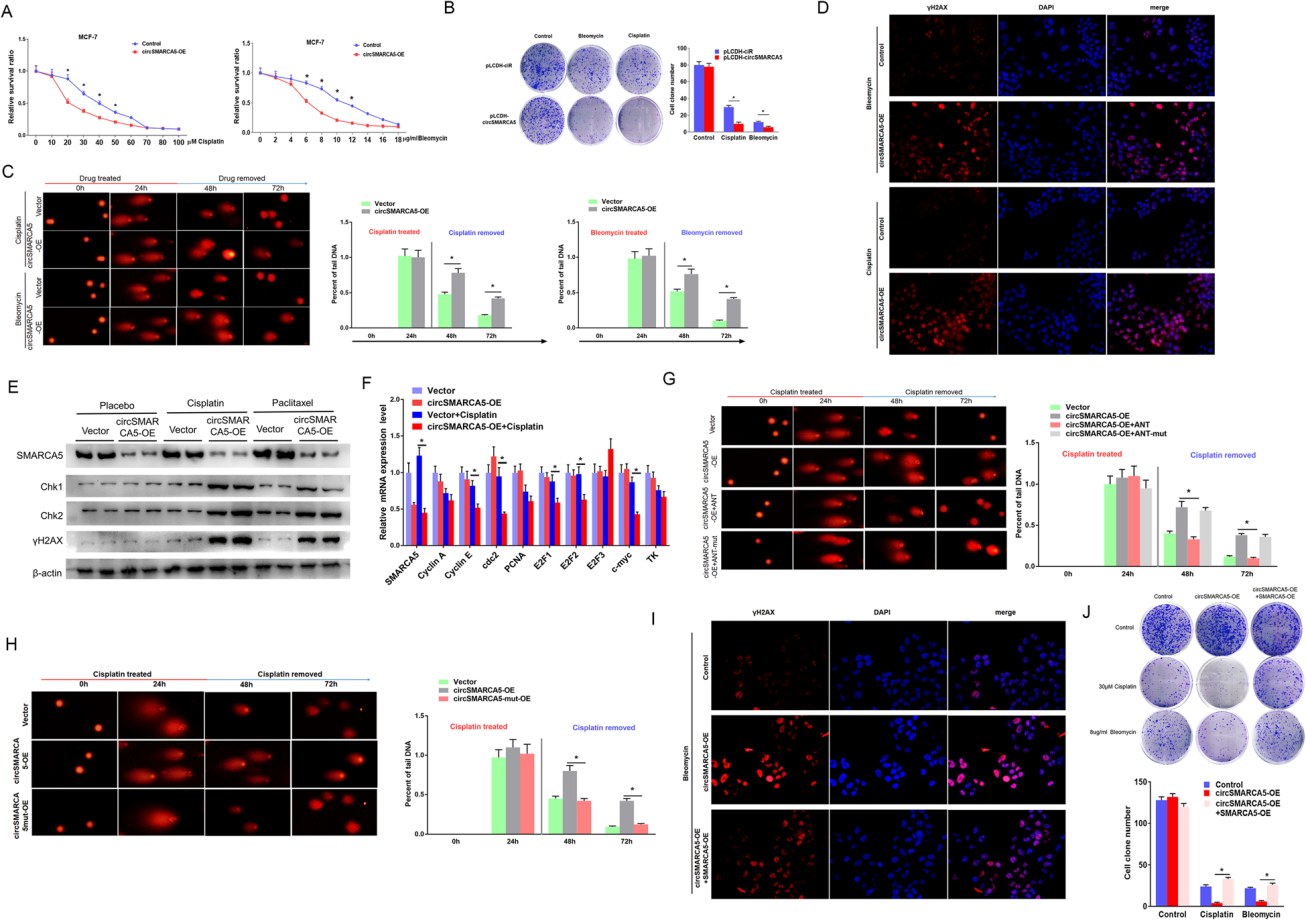
circSMARCA5 decreases DNA repair capacity. **a** circSMARCA5 increases sensitivity to cisplatin or bleomycin in MCF-7 cells. MCF-7 cells stably expressing control vector or pLCDH-circSMARCA5 were treated with cisplatin or bleomycin for 24 h, and CCK8 was used to measure cell viability. **b** Relative colony formation units of MCF-7 cells stably expressing control vector or pLCDH-circSMARCA5 treated with 20 μM cisplatin or 6 μg/ml bleomycin. After 24 hours, the drugs were replaced by fresh medium. The number of colonies was quantified. **c**, **d**, **e** Single cell gel electrophoresis (SCGE) assay indicating that circSMARCA5 overexpression inhibits cell recovery from DNA damage. MCF-7 cells stably expressing control vector or pLCDH-circSMARCA5 treated with 20 μM cisplatin or 6 μg/ml bleomycin. After incubation for 24 h, the cells were recovered with fresh medium for 24 or 48 hours and then collected for SCGE analysis (**c**), immunofluorescence assay using an anti-γH2AX antibody (**d**), and western blot assay with the indicated antibodies (**e**). **f** RT-qPCR assay showing the relative levels of several key cell cycle genes in MCF-7 cells stably expressing control vector or pLCDH-circSMARCA5 treated with DMSO or 20 μM cisplatin for 24 h and replaced with fresh medium for 72 h. “**” indicates *P* < 0.01. **g** SCGE assay showing that the cotransfection of ANT in circSMARCA5-overexpressing cells can restore the DNA repair capacity but the cotransfection of ANT-mut cannot. **h** SCGE assay showing that the overexpression of circSMARCA5 in MCF-7 cells can decrease DNA repair capacity but the overexpression of circSMARCA5-mut cannot. **i** SMARCA5 abrogates γH2AX levels induced by circSMARCA5. MCF-7 cells were infected with different combinations of lentivirus as indicated and treated with 6 μg/ml bleomycin. After incubation for 24 h, the cells were recovered with fresh medium for 48 hours and then collected for immunofluorescence assay using a γH2A antibody. **j** The cells as in **i** were treated with 30 μM cisplatin or 8 μg/ml bleomycin. After incubation for 24 h, the cells were recovered with fresh medium for 48 hours and then collected for colony formation assays. “*” represents *P* < 0.05

To explore the roles of circSMARCA5 in cancer progression, we overexpressed and depleted circSMARCA5 in MCF-7 cells by lentiviral vectors and then examined the effect of circSMARCA5 on cell proliferation, migration, and apoptosis. However, the results showed that both overexpressed and depleted circSMARCA5 had no effect on these three activities (Figure S9). Previous studies have indicated that SMARCA5 plays an important role in regulating the DNA repair process and maintaining the stability of the genome [22, 30–32]. Consistent with previous reports, SMARCA5 overexpression improved DNA repair capacity and reduced the expression of Chk1 and Chk2 after DNA damage repair (Figure S10A). Given that circSMARCA5 can promote the production of the truncated ΔSMARCA5 protein, we tested whether the truncated protein is also functional. The overexpression of Flag-ΔSMARCA5 had a minimal effect on the expression of Chk1 and Chk2 after DNA damage repair (Figure S10B), suggesting that ΔSMARCA5 is a nonfunctional protein product. We next assessed whether circSMARCA5 can affect the function of DNA damage repair capacity. CCK8 and clone formation assays revealed that circSMARCA5 overexpression increased sensitivity to cisplatin or bleomycin in MCF-7 cells (Fig. 7a, b). Next, MCF-7 cells were treated with the indicated concentration of cisplatin or bleomycin for 24 h and then the DNA damage was evaluated by single cell gel electrophoresis (SCGE) at 48 and 72 h. MCF-7 cells expressing circSMARCA5 showed significantly lower repair capacity than did control cells (Fig. 7c). In parallel, DNA damage was examined after 72 h of treatment with cisplatin or bleomycin by using an anti-γH2AX antibody. Consistent with the SCGE results, the γH2AX signal in MCF-7 cells expressing circSMARCA5 was significantly higher than that in MCF-7 cells, as evidenced by immunostaining (Fig. 7d). Consistently, cisplatin significantly enhanced the levels of DNA damage response proteins Chk1 and Chk2 in MCF-7 cells expressing circSMARCA5 (Fig. 7e), whereas several key cell-cycle genes were reduced specifically upon circSMARCA5 overexpression (Fig. 7f). To test whether circSMARCA5 R-loop formation is necessary for its DNA repair function, we transfected ANT or ANT-mut into circSMARCA5-expressing cells. The SCGE assay and γH2AX measurement showed that ANT significantly enhanced the DNA repair capacity, while ANT-mut had no effect on this activity (Fig. 7g and Figure S11). Furthermore, ANT significantly decreased the degree of colocalization between circSMARCA5 and its cognate DNA locus (Figure S12). In addition, unlike circSMARCA5, the overexpression of circSMARCA5-mut had little effect on the DNA repair rate (Fig. 7h and Figure S13A, B). Next, we determined whether SMARCA5 could mediate the effects of circSMARCA5 in preventing DNA damage repair. As shown in Fig. 7i, the γH2AX signal was much lower in circSMARCA5-expressing cells complemented with SMARCA5 than that in cells expressing circSMARCA5 alone. As expected, ΔSMARCA5 could not rescue the inhibition of DNA damage repair function induced by circSMARCA5 (Figure S13C, D). Moreover, the overexpression of SMARCA5 could significantly rescue the growth defects of cells expressing circSMARCA5, as demonstrated by a colony formation assay (Fig 7 j). Together, these results demonstrated the roles of circSMARCA5 in regulating the DNA repair process in MCF-7 cells.

### circSMARCA5 overexpression enhances the cisplatin response in breast cancer

**Fig. 8:**
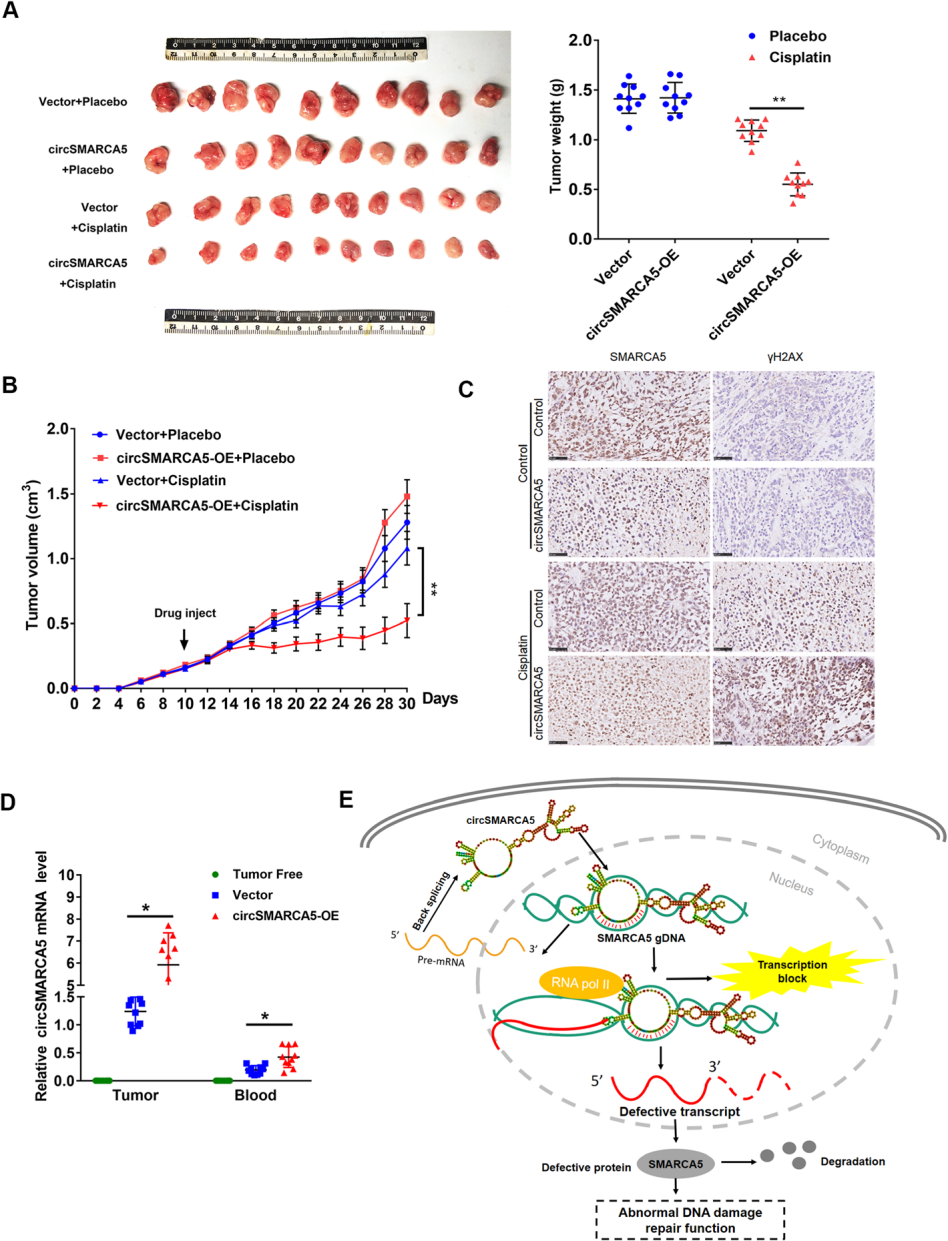
Figure 8. circSMARCA5 overexpression enhances the cisplatin response in breast cancer. **a** MCF-7 cells expressing control vector or pLCDH-circSMARCA5 were transplanted into mice. After the average tumor volume reached 0.3 cm3, the mice were injected with cisplatin or placebo. (Left) Representative images of the isolated tumors from injected mice. (Right) Tumor weight was calculated according to the formula provided in the Materials and Methods section. **b** After injection with cisplatin or placebo, the mean tumor volume was measured by calipers on the indicated days. **c** Representative immunofluorescence staining images (scale bars, 100 μm) and SMARCA5 and γH2AX expression in xenograft tumors derived from MCF-7 cells as in A. **d** RT-qPCR assay showing the relative levels of circSMARCA5 in the tumor tissues and blood of xenograft mice. The blood of tumor-free mice was treated as a control. “*” represents *P* < 0.05. **e** Proposed model for circSMARCA5 downregulation of its parent gene expression

To further evaluate the therapeutic potential of circSMARCA5 in breast cancer in vivo, we established circSMARCA5 overexpression clones in MCF-7 cells. As shown in Fig. 8a, the overexpression of circSMARCA5 efficiently enhanced the sensitivity of MCF-7 xenografts to concurrent cisplatin treatment (Fig. 8a, b). The overexpression of circSMARCA5 was confirmed by in situ hybridization and qPCR analysis (Fig. 8c), along with decreased SMARCA5 protein levels and increased γH2AX levels (Fig. 8d). In addition, qPCR analysis demonstrated that circSMARCA5 can be detected in the blood, suggesting that circSMARCA5 is a secretory molecule. Collectively, these data demonstrate that circSMARCA5 could serve as a potential therapeutic target to restore sensitivity to cisplatin therapy in breast cancer.

## Discussion

Previous studies have indicated that circRNAs have multiple functions in cancer development and progression [33–35]. In this study, we identified multiple expressed circRNAs in breast cancer samples and observed average higher abundance of circRNAs over their host genes in peripheral blood than tissues, which might contribute to the exploration of diagnostic biomarker for breast cancer. We then identified that circSMARCA5 is significantly decreased in breast cancer tissues using RNA-seq. More importantly, we define a critical role for circSMARCA5 in the regulation of DNA damage repair capacity and the drug sensitivity of breast cancer cells in vitro and in vivo through the negative regulation of its parent gene SMARCA5. These findings are of high clinical relevance because chemotherapy with cisplatin and bleomycin remains the standard of care in breast cancer [36–38]. Hence, the restoration of circSMARCA5 levels provides an approach to overcome treatment resistance in breast cancer patients.

SMARCA5, also known as SNF2H, is a member of the SWI/SNF chromatin-remodeling complex. During DNA damage repair processes, SMARCA5 is recruited to DNA damage sites where it induces the ubiquitination and phosphorylation of histone H2A, which facilitates chromatin remodeling and DNA damage repair [18, 19]. In this study, we show that circSMARCA5 expression resulted in the downregulation of SMARCA5, and the effect of circSMARCA5 overexpression on DNA repair capacity was reversed by concomitant SMARCA5 overexpression, suggesting that the effect of circSMARCA5 on DNA repair capacity is mediated through SMARCA5. circRNAs exert functions in various ways, such as forming an R-loop with DNA to regulate splicing and transcriptional pausing [17]. For example, circSEPALLATA3 regulates the splicing of its parent mRNA through R-loop formation [17]. In addition, circRNAs are a novel class of ceRNAs that sponge miRNAs, thus positively regulating gene expression [13, 14]. Additionally, circRNAs, such as exon-intron circRNAs, regulate gene expression through specific RNA-RNA interactions with U1 snRNA [39]. Furthermore, circRNAs also exert functions by binding to proteins and regulating their activities [40]. We identified one mechanism by which circSMARCA5 regulates the drug sensitivity of breast cancer cells to cisplatin and bleomycin through the downregulation of SMARC5. circSMARCA5 is recruited to its parent gene locus, leading to R-loop formation, transcription termination, nonfunctional truncated ΔSMARCA5 protein upregulation, and decreased SMARCA5 expression. This regulatory mechanism has also been verified in cervical cancer (Hela cells) (Figure S14). However, our evidence demonstrates that circSMARCA5 has no significant effect on the proliferation, migration and apoptosis of breast cancer cells, suggesting that this molecule functions in a cell-type and context-dependent manner. Notably, we provide evidence that circSMARCA5 plays a key role in the DNA repair pathway and drug resistance, making it a promising therapeutic target for breast cancers.

CircSMARC5 has been shown to be downregulated in hepatocellular carcinoma tissues, and its downregulation is associated with aggressive characteristics and unfavorable outcomes [25]. We also confirmed a low abundance of circSMARCA5 in breast cancer and renal cancer tissues compared with matched adjacent normal tissues.

Due to the features of circRNA, including stability, tissue specificity and abundance in bodily fluids, circRNAs may serve as potential biomarkers for various diseases [3, 41, 42]. Altogether, the findings in this study confirmed that the overexpression of circSMARCA5 was sufficient to improve the chemosensitivity of breast cancer cells in vitro and in vivo, indicating the important regulation mechanism of this circRNA in breast cancer. And our analysis revealed the negative correlation is also existed in other cancers, suggesting a general regulation of circSMARCA5 on host gene in transformed cells. Furthermore, we found that the ratio of circ-to-linear (expression of circRNA / linear host genes) of circSMARCA5 in the blood sample of breast cancer patients were significantly lower than health volunteers (Fig. 1c and S1B), which indicating that circSMARCA5 may serve as an indicator for the evaluation of breast cancer liquid biopsy. Further clinical research is needed to clarify whether circSMARCA5 is a useful biomarker in breast cancer.

## Conclusion

We observed average higher expression of circRNAs than their host genes in blood samples comparing to breast cancer and adjacent normal tissues. And for the first time, we validated the interaction between circRNA and its host gene DNA in cancer, through a regulation model of circRNA and the transcription of partial exons of host gene. Furthermore, we found circRNA could affect the sensitivity of tumor to chemotherapy drug and our result indicated this circRNA is involved in DNA damage repair in human cancer. Our results revealed a new regulatory mechanism for circRNA on its host gene and provided evidence that circSMARCA5 may serve as a therapeutic target for drug-resistant breast cancer patients.

## Materials and methods

### Cell culture and cell transfection

All cell lines, including 293 T, MCF-10A, MCF-7, SKBR3, BT474, T47D and MDA-MB-231, were purchased from the China Center for Type Culture Collection (CCTCC, Chinese Academy of Sciences, Shanghai, China). The cells were cultured according to the protocols from the American Type Culture Collection (ATCC; https://www.atcc.org/). Plasmid and phosphorylated deoxyribonucleic acid single strand transfection were carried out by Zlipo2000 (Zomanbio, Beijing, China) or liposomal transfection reagent (Yeasen, Shanghai, China).

### Sample collection

Breast cancer tissues, adjacent tissues and blood samples were collected from breast cancer patients treated at Zhongnan Hospital of Wuhan University (Wuhan, China) after written informed consent was obtained. One milliliter of EDTA blood from different breast cancer patients was collected before surgery. The blood samples were stored in Trizol at − 80 °C until use. In the RNA-seq study, 6 women (mean age: 52 years, range: 45–60 years) diagnosed with luminal-B subtype breast cancer were included. The amplification and overexpression of HER2 were found in 3 patients, while the other patients were negative in HER2 status. In addition, cancer and adjacent normal tissues and blood samples from 24 breast cancer patients with clinical pathologic information, and blood samples from 24 health volunteers were collected for the qPCR validation.

### Whole transcriptome sequencing

Total RNA was extracted from the tumor, adjacent normal tissue and blood samples of six breast cancer patients, followed by rRNA depletion from total RNA. Then, the RNA was reverse transcribed to cDNA and constructed into a strand-specific library. Illumina HiSeq XTen was performed for sequencing. All raw data can be accessed in the NCBI GEO database (https://www.ncbi.nlm.nih.gov/geo/query/acc.cgi?acc=GSE133998, accession code: wxmjuumulvsbbuf).

### Identification of circRNA expression

To identify the expression of circRNAs from RNA-Seq, one extensively used algorithm Circexplorer3 [43] were utilized for detecting the back-splice junction sites of circRNAs. And the fragments per billion mapped bases (FPB) of circRNA and its linear host gene were extracted to calculate the CIRCscore (FPBcirc/FPBlinear), which could indicate the expression ratio of circRNA to their host genes. Genome assembly GRCh37 and GENCODE (version 19) gene annotation were used. Total 8312 circRNAs were detected with expression across all six patients.

### Northern blot analysis

Northern blot (NB) analysis was performed according to the manufacturer’s instructions (DIG Northern Starter Kit, Roche). Digoxigenin (DIG)-labeled riboprobes were transcribed by DIG RNA labeling Mix (Roche, USA) and the HiScribe T7 In Vitro Transcription Kit (NEB, USA), and then purified by phenol-chloroform extraction and ethanol precipitation. Ten micrograms of total RNA or 1 mg of in vitro synthesized linear or circRNA was electrophoresed on a denaturing urea polyacrylamide gel, transferred to a Hybond-N+ membrane (GE, USA) by a positive capillary transfer system and UV-crosslinked as a standard protocol. The membrane was then hybridized with specific DIG-labeled riboRNA probes. NB probes are listed in Table S2.

### DNA and RNA fluorescence in situ hybridization (FISH)

The cells were fixed with 4% paraformaldehyde. After permeabilization with proteinase K pretreatment, the DNA was denatured with 2X SSC solution containing 70% formamide at 72 °C and then incubated for 12 h at 37 °C in hybrid solution [50% formamide, 2X SSC, 0.25 mg/mL *Escherichia coli* transfer RNA, 0.1 mg/mL salmon sperm DNA (Sigma, USA), 2.5 mg/mL BSA (Sigma, USA), and DIG- or biotin-labeled 100 pM probes transcribed by T7 In Vitro Transcription Kit (NEB, USA)]. After washing with hybrid solution supplemented with RNase A to remove excessive probes, the cells were subjected to indirect immunofluorescence using FITC/AF594 coupled with anti-DIG antibody or FITC/AF594 coupled with streptavidin. The nuclei were stained with DAPI. The probes are listed in Table S2.

### Plasmid constructs

The sequence for exons 15–16 of SMARCA5 was PCR amplified using primers F (5′-CGGAATTCTGAAATATGCTATCTTACAG GGAGGCTTGTGGATCAGAAT.

CTG-3′) and R (5′-CGGGATCCTCAAGAAAAAATATATTCACCTTTTGTTTTTC.

TCTATAGTCTCTCC − 3′), digested by EcoRI and BamHI, and ligated into pLCDH-ciR (Geneseed, China) and pLCDH-ciR-GFPmut (GFP mutated by site-directed mutagenesis) to create two circSMARCA5-overexpressing plasmids. The pCMV-DsRed-T2A-MSC-EGFP reporter vector was constructed by cloning the coding region of DsRed and T2A into psi-EGFP-N1(Addgene, USA). Exons 13–14, 15–16 and 13–16 of SMARCA5 were PCR amplified and cloned into the pCMV-DsRed-T2A-MSC-EGFP reporter by in-Fusion cloning. To overexpress SMARCA5 and ΔSMARCA5 protein, the SMARCA5 complete and defective transcript sequences were cloned into pFLAG-CMV2 (Addgene, USA). Gene-specific shRNA target sequences were synthesized and cloned into the pLKO.1 plasmid. The primers for making these constructs are provided in Table S3.

### RNA secondary structure prediction

The circSMARCA5 complete sequence was input into the RNAfold web server (http://rna.tbi.univie.ac.at/cgi-bin/RNAWebSuite/RNAfold.cgi). The minimum free energy structure and thermodynamic ensemble structure were predicted by this website.

### The 3′ rapid amplification of cDNA ends (3′ RACE)

The total RNA was extracted from cells, and the integrity was verified. Oligo dT with a tag sequence was used for reverse transcription. The 3′ end sequences of mRNA were PCR amplified using primers located in SMARCA5 exon 2 and the tag sequence. Fragments with different expression levels were identified by one-generation sequencing. All primers are listed in Table S3.

### R-loop dot-blotting

Dot-blotting was performed using S9.6 monoclonal antibody according to previously published protocols [17, 44]. DNase I, RNase H and RNase R (Epicentre Technologies) treatments were performed essentially as described using a standard hybridization buffer (10 mM Tris-HCl, pH 7.5, 50 mM NaCl, 0.1 mM EDTA). RNA for exons 13–14, 15–16 and 15–16 mutation templates of SMARCA5 were transcribed with a T7 RNA Polymerase kit (NEB, USA) and circularized by splint ligation with T4 RNA ligase with circRNAs purified from linear RNAs by RNase R digestion. DNA templates, including SMARCA5 exons 13–14, 15–16, 13–16 and their mutation templates, were amplified by PCR. The strength of the R-loops was assessed by increasing the hybridization stringency through a stepwise decrease in ionic strength from 80 to 10 mM NaCl. The dot-blot experiments were repeated three times, with quantification performed with Image Lab software ver 5.2.1 (mean, background normalized values presented).

### Drip

DRIP was carried out as described [45] with some modifications. In brief, 40 μg of genomic DNA was extracted from cells by PCI (phenol:chloroform:isoamyl alcohol = 24:23:1), with 20 μg/ml proteinase K (Sigma, USA) and 20 μg/ml RNase A (Sigma, USA) applied to degrade proteins and RNA in the genome, respectively. The DNA was digested with 6 U EcoRI, EcoRV, XbaI, BamHI, and SspI (NEB, USA) at 37 °C overnight or treated with 0.4 U/μl RNase H (Diamond, China) as a negative control for R-loop validation. The purified DNA fragment at 10 μg was incubated with 10 μg S9.6 antibody (Kerafast, USA) at 4 °C for 2 h in 0.2 ml IP buffer (20 mM HEPES-KOH (pH 7.5), 150 mM NaCl, 10 mM MgCl2, 0.5% Triton X-100), with mouse IgG (CST, USA) as a control. The mixture was incubated with 20 μg protein A/G agarose beads (GE, USA) at 4 °C overnight. The beads were washed three times with IP buffer and treated with 20 ng proteinase K at 45 °C for 30 min. The DNA was purified and applied by PCR or qPCR. The primers are listed in Table S3.

### Mass spectrum analysis

To detect ΔSMARCA5, a defective protein of SMARCA5, SDS-PAGE of the target protein molecule weight region was used to analyze the protein mass spectrum. In brief, MCF-7 cells overexpressing circSMARCA5 were treated with 30 μM MG132 for 24 h. The cells were harvested, and the total protein was extracted for SDS-PAGE. When the 90 kd band and 110 kD protein marker band were sufficiently separated, the electrophoresis was stopped, and the SDS-PAGE gel was excised between 70 and 90 kD for analysis by mass spectrometry. Mass spectrometry data were analyzed by Mascot 2.3 and aligned with UniProt human_20190102_177661.fasta protein sequence data.

### Real-time quantitative PCR (qPCR)

For RT-qPCR, total RNA was extracted from breast cancer tissues and adjacent tissues, rRNA was depleted, and RNase R was used to digest the linear RNA. qPCR was carried out as described [46]. In brief, total RNA was extracted by TRIzol reagent (Invitrogen, Carlsbad, CA), and cDNA was synthesized by random primers or oligo-dT (Yeasen ,China). qPCR was performed on a CFX 96 real-time PCR system using SYBR Green Real-time PCR Master Mix (Bio-Rad, USA). The expression levels of target genes were normalized by 2^-∆∆Ct^ with GAPDH and β-actin as references. Each experiment was repeated three times. The primers used in qPCR are listed in Table S3.

### Single cell gel electrophoresis

A comet assay was carried out as described [47]. In brief, 1 × 10^5^ cells were mixed with 1 ml agarose (0.8% agarose in PBS). The suspension of agarose cells was dripped onto the slide to form a uniform film. The slide was incubated at 4 °C for 10 min, and then placed in a slide box, followed by the addition of e precooled cracking liquid (NaCl 14.6 g, 100 mm EDTA 500 μl, 0.4 M Tris 250 μl, Triton X 100 ml, DMSO 10 ml, adjust solution pH to 10 with NaOH). Avoiding light and using a constant 20 V voltage, electrophoresis was performed for 30 min. The slide was removed after electrophoresis and soaked 2 times in 0.4 m Tris solution (formula 9.69 g Tris with constant volume to 200 ml, and the solution pH was adjusted to 7.5 ml) for 5 min each. Then, the slides were removed and stained in 2 μg/ml EB or acridine orange solution for 5 min, washed twice with pure water for 10 min, and observed under a fluorescence microscope. The cell trailing rate and mean tail length were analyzed and calculated by CASP software.

### Xenografts in nude mice

Female BALB/cnu/nu nude mice, 28–30 days old and weighing 16–18 g, were maintained under sterile conditions and fed sterile feed and water. The nude mice were divided into 4 groups, with 6 mice in each group. Each mouse was injected subcutaneously with prepared cells (1 × 10^7^) at a single site. The tumor volume was measured with calipers and calculated using the formula VT = 1/2(L**╳** W **╳** W) (L: the maximum of tumor; W: the minimum length of tumor). When the tumor volume was 0.1 cm^3^, two groups were injected with cisplatin (10 mg/kg/day), and the other two groups were given 1X PBS as a placebo. When the tumor of the control group grew to 200 μl, all mice were sacrificed, and the xenograft was removed, weighed and measured to determine tumor volume. All operations are carried out following the Guidelines for Animal Experimentation of Wuhan University. Our protocol was approved by the Ethics Committee for Animal Experimentation and was performed on the basis of the Guidelines for Animal Experimentation of Wuhan University and the National Institute of Health Guide for the Care and Use of Laboratory Animals.

### Immunohistochemistry

Tissue sections (5 μm) were deparaffinized in a gradient dilution of xylene and then hydrated in a gradient dilution of absolute ethanol. Subsequently, endogenous peroxidase activity was blocked by the freshly prepared solution of 3% H_2_O_2_ for 10 min at room temperature. After antigen retrieval was performed in 0.02 M PBS buffer (pH 7.2–7.6), 5% BSA blocking was conducted at 37 °C for 30 min. After washing with PBS, the primary antibodies (Including anti-Smarca5, diluted 1:200, R; anti-γH2AX, diluted 1:100, R) were incubated at 4 °C overnight. The secondary antibody (diluted 1:1000, R) was incubated for 2 h at room temperature. Finally, the slides were stained with DAB and counterstained with hematoxylin. A Panoramic MIDI automatic digital slide scanner (3DHISTECH Ltd., Budapest, Hungary) was used for image processing and quantification. The expression levels of the target proteins (Smarca5, γH2AX) in each tissue sample were examined based on the intensity of immunohistochemical staining.

### Flow cytometry

The cell cycle was analyzed by flow cytometry. The Ex13–14, Ex15–16, and Ex13–16 sequences were inserted into a series of sequences of PCMV-DsRed-T2A-MSC-EGFP from the MSC site in the form of recombinant vectors. After 48 h of transfection in the experimental group, the three recombinant vectors were stably transfected into a cell line expressing circSMARCA5 and a control cell line. The cells were harvested with trypsin, fully resuspended in a single cell suspension, and the negative control tubes and specimen tubes were sequenced according to the order of the samples, and the fluorescence was detected by flow cytometry. The two detection channels used were FITC-A and PE- A, respectively. For cell cycle, the cells were stained with PI Cell cycle kit (Yeasen, China) and detected by PE channel, which has the same excitation wavelength with PI. For the BrdU staining, 107 cells were labeled with 10 μM BrdU for 2 h at 37 °C. Cells were fixed and intracellularly stain with Anti-BrdU-APC antibody. The cells were counterstained with PI, and the stained cells were analyzed by flow cytometry. A total of 10,000 cells were detected, and the number of cells in each fluorescent region was analyzed. For cell transcription analysis, cells transfected with pDsRed-C1 or pEGFP-C1 (Addgene, USA) were used as channel gates to analyze the proportion of cells in each region.

### Chromatin Immunoprecipitation (ChIP)

ChIP was carried out as described [48]. In brief, the cells were crosslinked with formaldehyde at a final concentration of 1% for 10 min, and then glycine was added to a final concentration of 125 mM and incubated with shaking for 5 min. The cells were resuspended in cell lysis buffer (5 mM PIPES (pH 8.0), 85 mM KCl, 0.5% Nonidet P-40) and incubated on ice for 10 min to allow the release of nuclei. The nuclear pellet was resuspended in MNase digestion buffer (0.32 M sucrose, 50 mM Tris-Cl (pH 7.6), 4 mM MgCl_2_, 1 mM CaCl_2_, 0.1 mM PMSF) for 1 h on ice. EDTA was added to 50 mM to stop the digestion. The supernatant was then sonicated. After measuring the DNA concentration, fragmented chromatin (300–700 bp) was diluted with dilution buffer (10 mM Tris-Cl (pH 8.0), 0.5 mM EGTA, 1% Triton X-100, 140 mM NaCl) supplemented with 1 mM PMSF. The cleared supernatant was incubated with 2 μg of rabbit anti-Rpb1 antibody (RNA polymerase II, CST, Danvers, MA, USA) or 2 μg of anti-rabbit IgG (CST, Danvers, MA, USA) on a rocker overnight. After adding 40 μL protein A/G Dyna-beads (Life Technologies, Carlsbad, CA), the reactions were incubated for 2 h at 4 °C. The beads were washed with cell lysis buffer, low-salt wash buffer (200 mM NaCl, 50 mM Tris-HCl (pH 8.0), 5 mM MgCl_2_, 1% Triton X-100), high-salt wash buffer (50 mM HEPES (pH 7.9), 500 mM NaCl, 1 mM EDTA, 0.1% SDS, 1% Triton X-100, 0.1% deoxycholate), LiCl buffer (250 mM LiCl, 100 mM Tris-HCl (pH 8.0), 5 mM EDTA, 0.5% Na-deoxycholate, 0.5% Triton X-100) and TE buffer (100 mM Tris-Cl (pH 7.5). The bound DNA was eluted and reverse-crosslinked using elution buffer (50 mM Tris-Cl (pH 8.0), 10 mM EDTA, 1% SDS, 20 ng proteinase K) at 65 °C overnight. The DNA was purified and analyzed by real-time qPCR assay. The specific primers (Table S3) were applied.

### Cycloheximide chase assay

The cycloheximide assay was carried out as described [49]. MCF-7 cells were cultured in 6-well plates at a density of 1 × 10^5^ cells/well and then transfected with plasmid pFLAG-CMV-SMARCA5 or FLAG-CMV-ΔSMARCA5 during the logarithmic phase. Forty-eight hours after transfection, cycloheximide was added to the medium at a final concentration of 50 μg/ml. MCF-7 cells were harvested after treatment with cycloheximide for 0.5, 1, 2, 4, or 8 h. A western blot assay was applied to detect the expression level of SMRCA5 or ΔSMARCA5. A mAb against flag (AB clonal, Cambridge, MA, USA, dilution 1:1000) was used to detect SMARCA5 expression, and the rAb SMARCA5-N-terminal region (ABclonal, USA, dilution 1:1000) was used to detect ΔSMARCA5 expression.

### Phosphorthioate modification of DNA

To detect the target sequence of circSMARCA5 binding to exon 15–16 DNA, phosphorylated DNA sequences were applied. In brief, the DNA sequence 5′-ATTTGAAGCATTTCATCTTTCCCAATTTTGTT-3′ was synthesized with phosphorthioate modification of three bases at the 5′ and 3′ ends and named ANT. The mutation of ANT was named ANT-mut, with cytosine converted to guanine. The ANT phosphorthioate-modified DNA was applied to a dot-blot assay at a concentration of 10 μg/ml to examine the binding ability to circSMARCA5. Then, 10 pmol of ANT phosphorthioate-modified DNA was transfected into MCF-7 cells in each well of a six-well plate for 48 h, and the SMARCA5 protein expression level was detected by western blotting.

### Immunofluorescence

The cells were inoculated on the slide and cultured in a 24-well plate. After DNA damage drugs (cisplatin or bleomycin) were applied for 24 h, the cells were washed with PBS. Subsequently, the cells were fixed with 4% paraformaldehyde and then permeabilized with 0.5% Triton X-100 in PBS for 10 min at room temperature. After treating the cells with 5% BSA for 30 min at room temperature, the slides were incubated with antibody (anti-SMARCA5 or anti-γH2AX) overnight at 4 °C. After washing with 1X PBST (0.1% Tween 20 in PBS) 3 times, the cells were subjected to indirect immunofluorescence using a fluorescence-labeled antibody (rabbit 594/488, Proteintech, USA). The nuclei were stained with DAPI. The images were taken using a fluorescence microscope (Olympus, Japan).

### Nuclear and cytoplasmic separation

The cells were collected and resuspended in cell lysate buffer (1% NP40, 5 nM EDTA, and 0.5% sodium deoxycholate) for 5 min, followed by centrifugation at 4000 rpm for 1 min at 4 °C. The cytoplasmic proteins were present in the supernatant. The pellet was washed with cell lysate buffer for 10 min at 4 °C and then centrifuged for 5 min at 4 °C to collect the nuclei.

### Statistical analyses

All statistical analysis was done with the software Graphpad Prism (GraphPad Software, La Jolla, CA) and R packages (www.r-project.org).

## Supplementary information

**Additional file 1: Figure S1.** The expression level of SMARCA5 and circSMARCA5 in blood and tisssues sample of breast cancer patients health volunteers and tisssues sample of renal cancer. **Figure S2.** RNA FISH showing circSMARCA5 was mainly expressed in the nucleus. **Figure S3.** circSMARCA5 decreases the expression of SMARCA5 in MCF-7 cells. **Figure S4.** The protein level of SMARCA5 in breast cancer and adjacent normal tissues. **Figure S5.** The expression correlation of circSMARCA5 and SMARCA5in different tumors. **Figure S6.** circSMARCA5 overexpression downregulated the protein levels of SMARCA5 while upregulating the truncated SMARCA5 (ΔSMARCA5) protein levels. **Figure S7.** Fragmented genomic DNA for DRIP-qPCR experiments. **Figure S8.** The secondary structure of circSMARCA5 and circSMARCA5-mut. **Figure S9.** circSMARCA5 has no significant effect on the proliferation and migration ability of breast cancer cells. **Figure S10.** The truncated protein ΔSMARCA5 is a nonfunctional protein product. **Figure S11.** Immunofluorescence assay using a γH2AX antibody showing that the cotransfection of ANT in circSMARCA5-overexpressing cells can abrogate γH2AX levels inducedby circSMARCA5 but the cotransfection of ANT-mut cannot. **Figure S12.** ANT significantly decreased the degree of colocalization between circSMARCA5 and its cognate DNA locus. **Figure S13.** (A) MCF-7 cells expressing control vector, circSMARCA5 or circSMARCA5-mut were treated with cisplatin or bleomycin in concentration gradient for 24 h, and CCK8 was used to measure cell viability. **Figure S14.** circSMARCA5 downregulate SMARCA5 and suppress DNA damage repair in Hela cell.

**Additional file 2: Table S1.** The ratio of circ-to-linear of circSMARCA5 (expression of circRNA / linear host genes) and clinical pathologic characteristics of breast cancer.

**Additional file 3: Table S2.** Probes for circRNA detection.

**Additional file 4: Table S3.** Probe for transcribed RNA and expression plasmid.

## Data Availability

All RNASeq raw data can be accessed in the NCBI GEO database (https://www.ncbi.nlm.nih.gov/geo/query/acc.cgi?acc=GSE133998, accession code: wxmjuumulvsbbuf).

## Abbreviations

circRNA: circular RNA
RBP: RNA binding protein;
RNA-Seq: RNA sequencing
qPCR: Real-time quantitative polymerase chain reaction
HER2: Human epidermal growth factor receptor 2
ISH: In situ hybridization
IHC: Immunohistochemistry
IF: Immunofluorescence
RIP: RNA immunoprecipitation
ChIP: Chromatin Immunoprecipitation
DRIP: DNA-RNA immunoprecipitation
NB: Northern blot
FISH: Fluorescence In Situ Hybridization
3’RACE: The 3′ rapid amplification of cDNA ends
MS: Mass Spectrum
