# Supplementary material for: CircRNA inhibits DNA damage repair by interacting with host gene
Source: Mol Cancer. 2020 Aug 24;19:128. doi: 10.1186/s12943-020-01246-x (PMC7446195; doi:10.1186/s12943-020-01246-x)
Supplement: Supplementary file 1 — Additional file 1: Figure S1. The expression level of SMARCA5 and circSMARCA5 in blood and tisssues sample of breast cancer patients health volunteers and tisssues sample of renal cancer. Figure S2. RNA FISH showing circSMARCA5 was mainly expressed in the nucleus. Figure S3. circSMARCA5 decreases the expression of SMARCA5 in MCF-7 cells. Figure S4. The protein level of SMARCA5 in breast cancer and adjacent normal tissues. Figure S5. The expression correlation of circSMARCA5 and SMARCA5in different tumors. Figure S6. circSMARCA5 overexpression downregulated the protein levels of SMARCA5 while upregulating the truncated SMARCA5 (ΔSMARCA5) protein levels. Figure S7. Fragmented genomic DNA for DRIP-qPCR experiments. Figure S8. The secondary structure of circSMARCA5 and circSMARCA5-mut. Figure S9. circSMARCA5 has no significant effect on the proliferation and migration ability of breast cancer cells. Figure S10. The truncated protein ΔSMARCA5 is a nonfunctional protein product. Figure S11. Immunofluorescence assay using a γH2AX antibody showing that the cotransfection of ANT in circSMARCA5-overexpressing cells can abrogate γH2AX levels inducedby circSMARCA5 but the cotransfection of ANT-mut cannot. Figure S12. ANT significantly decreased the degree of colocalization between circSMARCA5 and its cognate DNA locus. Figure S13. (A) MCF-7 cells expressing control vector, circSMARCA5 or circSMARCA5-mut were treated with cisplatin or bleomycin in concentration gradient for 24 h, and CCK8 was used to measure cell viability. Figure S14. circSMARCA5 downregulate SMARCA5 and suppress DNA damage repair in Hela cell. [file 12943_2020_1246_MOESM1_ESM.pdf]

Supplemental Figures

Figure S1

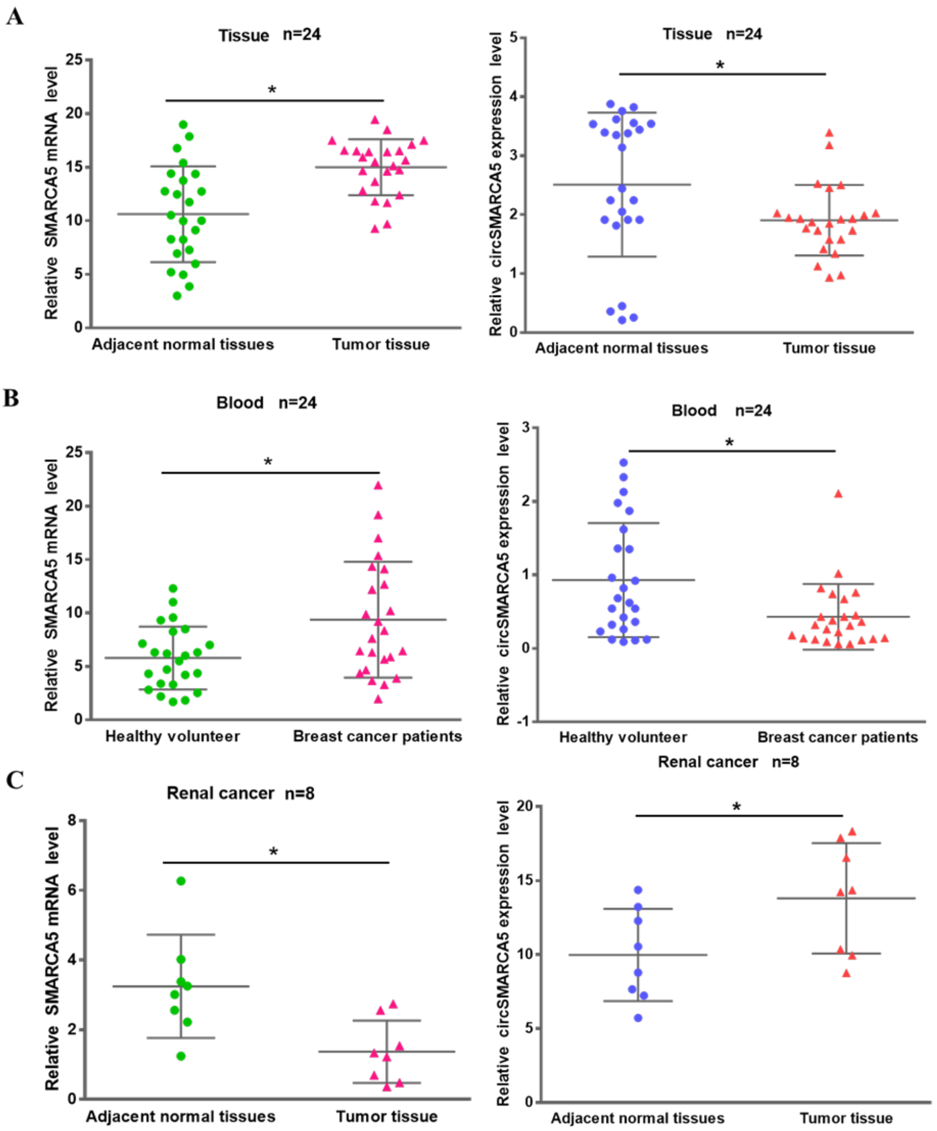

Figure S1 The expression level of SMARCA5 and circSMARCA5 in blood and tisssues sample of breast cancer patients health volunteers and tisssues sample of renal cancer. (**A**) The expression level of SMARCA5 and circSMARCA5 in breast cancer and normal adjacent tissues. Total RNA were extracted from cancer and adjacent normal tissues of 24 breast cancer patients, and the expression level was detected by RT-qPCR. (**B**) The expression level of SMARCA5 and circSMARCA5 in blood sample of breast cancer patients and health volunteers. Total RNA from blood sample of breast cancer patients and health volunteers was extracted and detected by RT-qPCR. (**C**) The expression level and ratio of circ-to-linear of circSMARCA5 in renal cancer and normal adjacent tissues. Total RNA were extracted from cancer and adjacent normal tissues of 8 renal cancer patients, and the expression level was detected by RT-qPCR. The expression level was normalized with β-actin as reference. *: $P < 0.05$ was considered statistically significant.

Figure S2

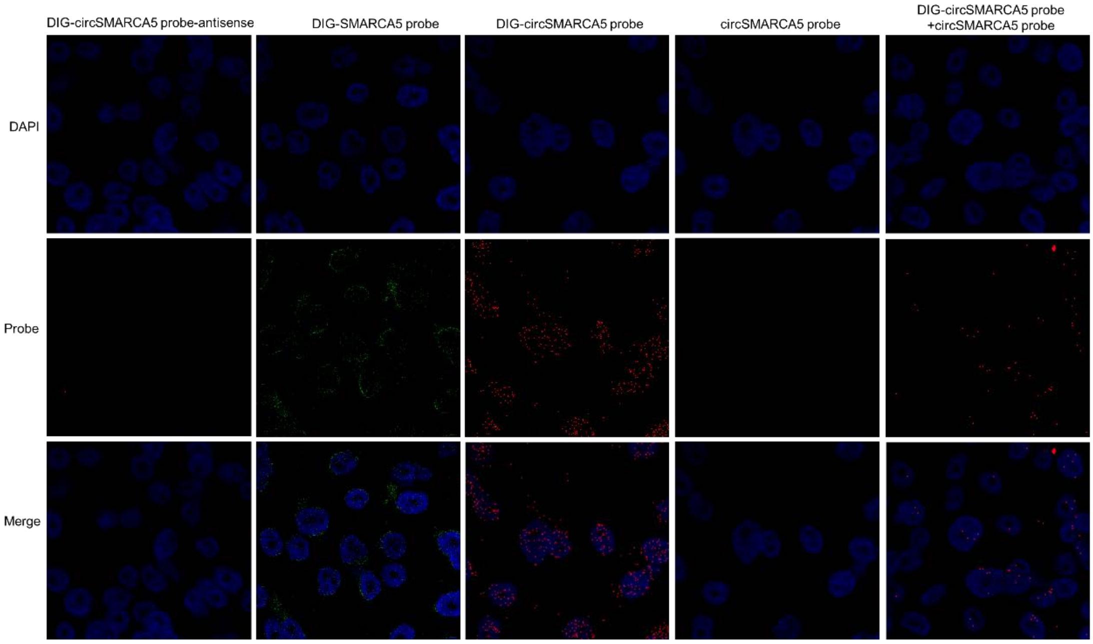

Figure S2. RNA FISH showing circSMARCA5 was mainly expressed in the nucleus. To illustrate the specificity of the circSMARCA5 probe, we set up the antisense strand probe and the mRNA probe of SMARCA5 as a control. To demonstrate that the fluorescent signal was from the labeled DIG-circSMARCA5 probe, the circSMARCA5 probe without DIG was used for the competitive binding assay. The results showed that the content of fluorescent probe in the nucleus of the competition probe group was significantly reduced, which indicated that the fluorescent signal came from the DIG-labeled probe.

Figure S3

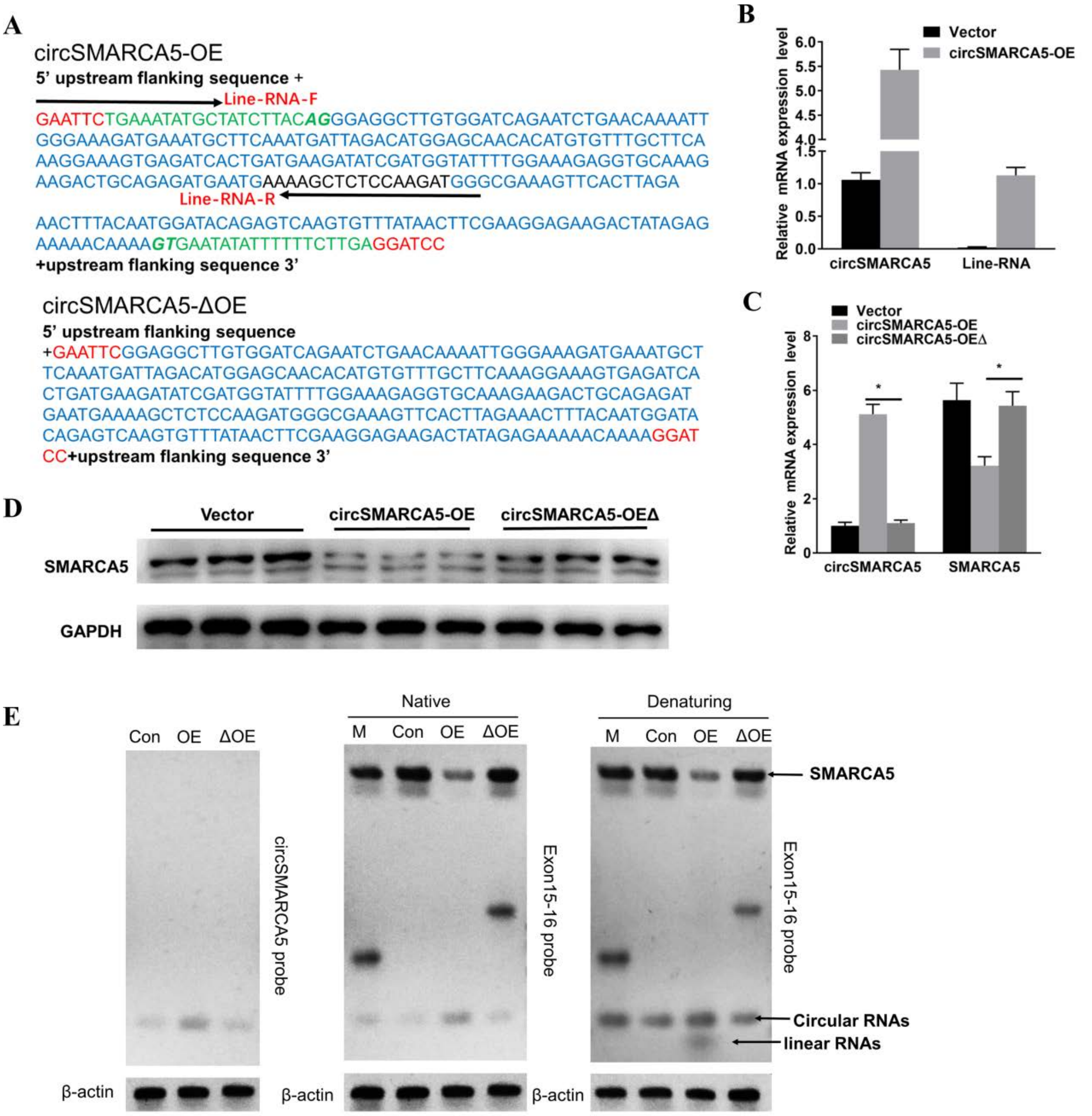

Figure S3. circSMARCA5 decreases the expression of SMARCA5 in MCF-7 cells. (**A**) Schematic illustration showing the overexpression vector for circSMARCA5 circRNA (circSMARCA5-OE) and exon 15-16 liner RNA (circSMARCA5-ΔOE cannot produce circRNA duo to a lack of the auxiliary looped splicing sequence). The restriction sitesare marked in red, the sequence of exons 15-16 is marked in blue, and the auxiliary looped splicing sequence is marked in green. Line-RNA-F and Line-RNA-R were primers for amplifying linear fragments. (**B**) RT-qPCR assay showing the circSMARCA5 circRNA and liner RNA in MCF-7 cells expressing circSMARCA5 overexpression (circSMARCA5-OE) or circSMARCA5-ΔOE. (**C**-**E**) MCF-7 cells were infected with lentiviruses expressing circSMARCA5-OE or circSMARCA5-ΔOE. RT-qPCR (**C**), western blotting (**D**) and northern blotting (**E**) were performed to evaluate the expression of circSMARCA5 and/or its parent SMARCA5 gene. Actin was used as an internal control.

Figure S4

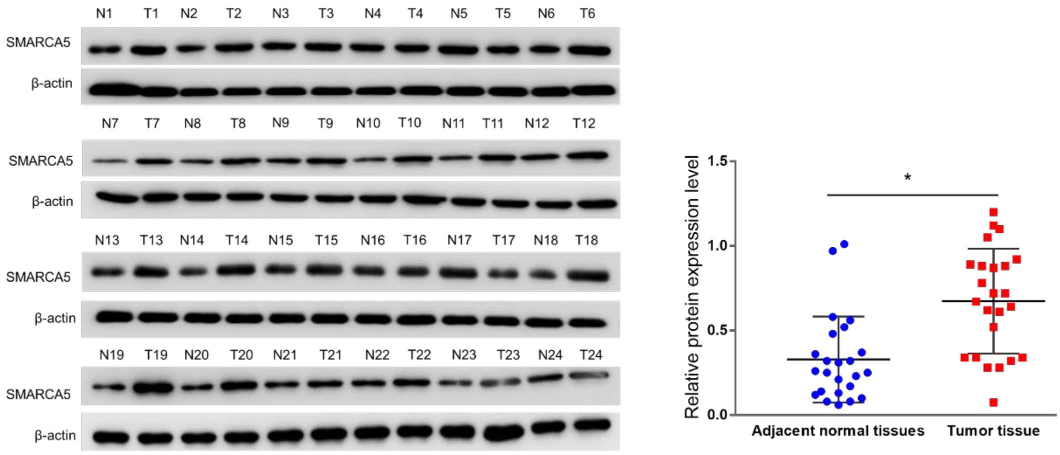

Figure S4. The protein level of SMARCA5 in breast cancer and adjacent normal tissues. The breast cancer tissue and adjacent normal tissue protein of 24 breast cancer patients were extracted. The protein expression level was detected by western blot. The expression level was normalized with β-actin as reference.*: $P < 0.05$ was considered statistically significant.

Figure S5

Cohort	Pearson	p-value	n
BRCA	-0.67511	0.018339127	30
PRAD	-0.55918	0.029677206	32
SARC	0.301779	0.034497308	27
BLCA	-0.6741	0.01948684	10
LUNG	-0.6933	0.0370919783	13
HNSC	0.00559	0.006858195	12

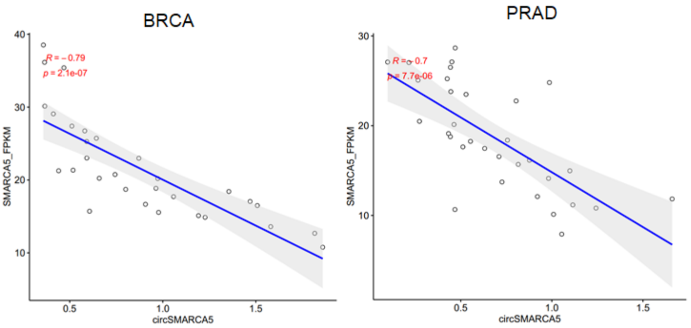

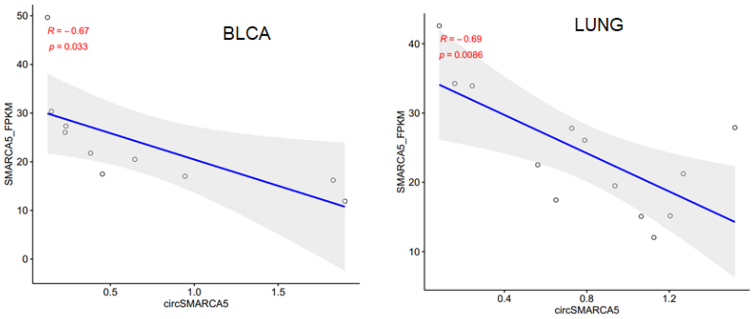

Figure S5. The expression correlation of circSMARCA5 and SMARCA5in different tumors. The reads of circSMARCA5 and FPKM of SMARCA5 were downloaded from MiOncoCirc (http://mioncocirc.github.io) database. Then the reads of circSMARCA5 were normalized by total mapped reads. The expression correlation is calculated by Pearson coefficient. Pearson coefficient is less than -0.5, p-value <0.05 To ensure the correlation results are reasonable, we picked up 6 cancer types with more than 10 samples and performed the pearson correlation. The results indicated a significant negative correlation (r<-0.5, P<0.05) between the expression levels of circSMARCA5 and SMARCA5 in breast cancer (BRCA), bladder cancer (BLCA), prostate cancer (PRAD) and lung cancer (LUNG), except sarcoma (SARC) and head and neck cancer (HNSC).

Figure S6

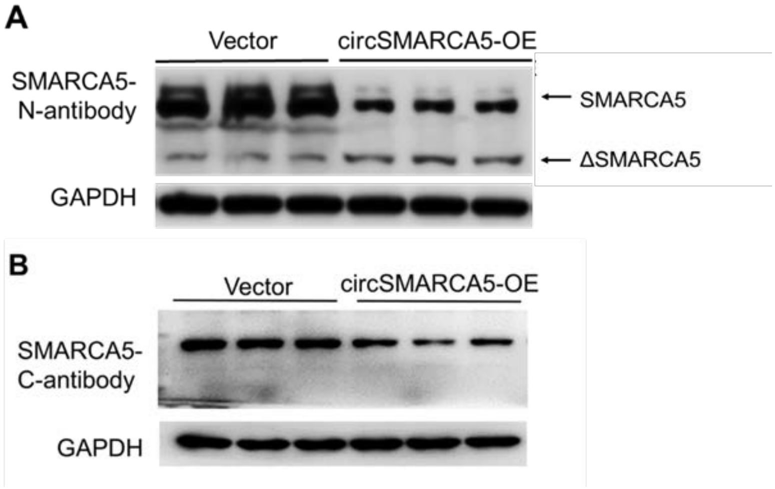

Figure S6. circSMARCA5 overexpression downregulated the protein levels of SMARCA5 while upregulating the truncated SMARCA5 (ΔSMARCA5) protein levels. (**A**) Western blot assay showing thatcircSMARCA5 promotes the production of C terminal truncated SMARCA5 (ΔSMARCA5) protein. The expression levels of full-length and truncated protein were analyzed using an antibody targeting the N terminus of the SMARCA5 protein. (**B**) The expression levels of full-length were analyzed using an antibody targeting the C-terminus of the SMARCA5 protein.

Figure S7

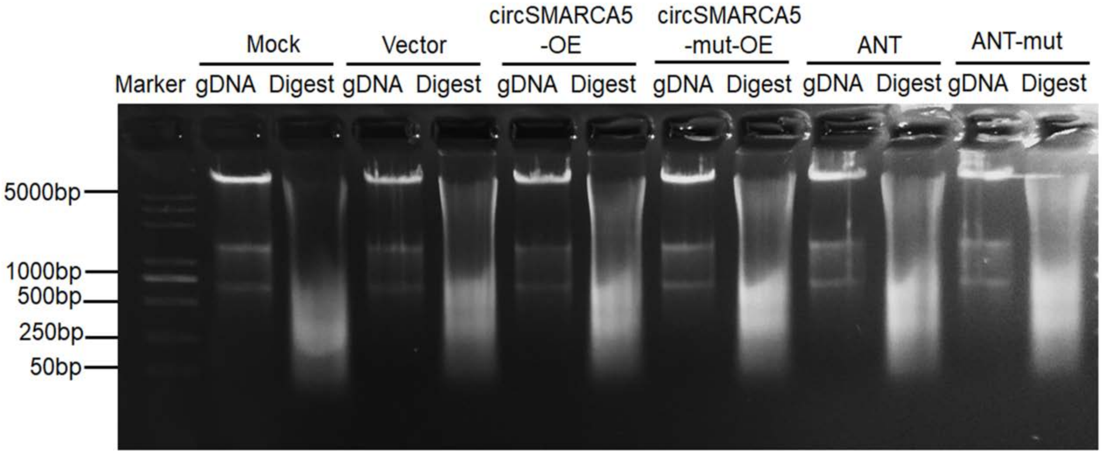

Figure S7. Fragmented genomic DNA for DRIP-qPCR experiments. To improve the resolution of DRIP-qPCR, the genomic DNA was fragmented with a variety of restriction enzymes (EcoRI, Hind III, Xhol I, Xbal I and SspI) to cleave the genomic DNA.

Figure S8

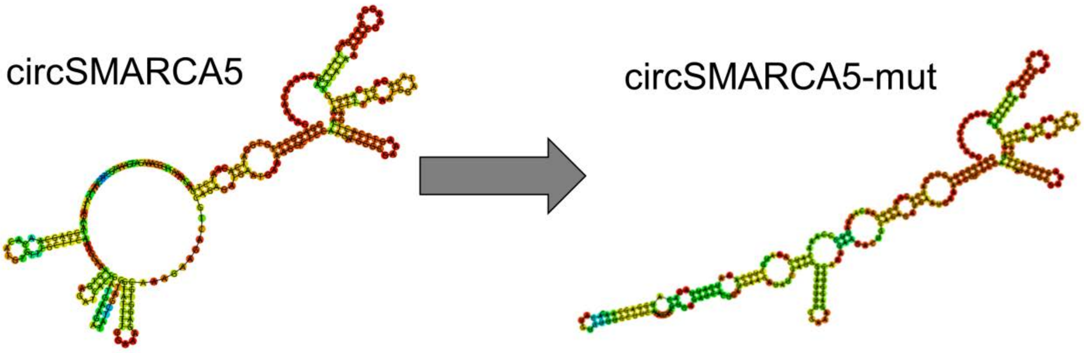

Figure S8. The secondary structure of circSMARCA5 and circSMARCA5-mut.

Figure S9

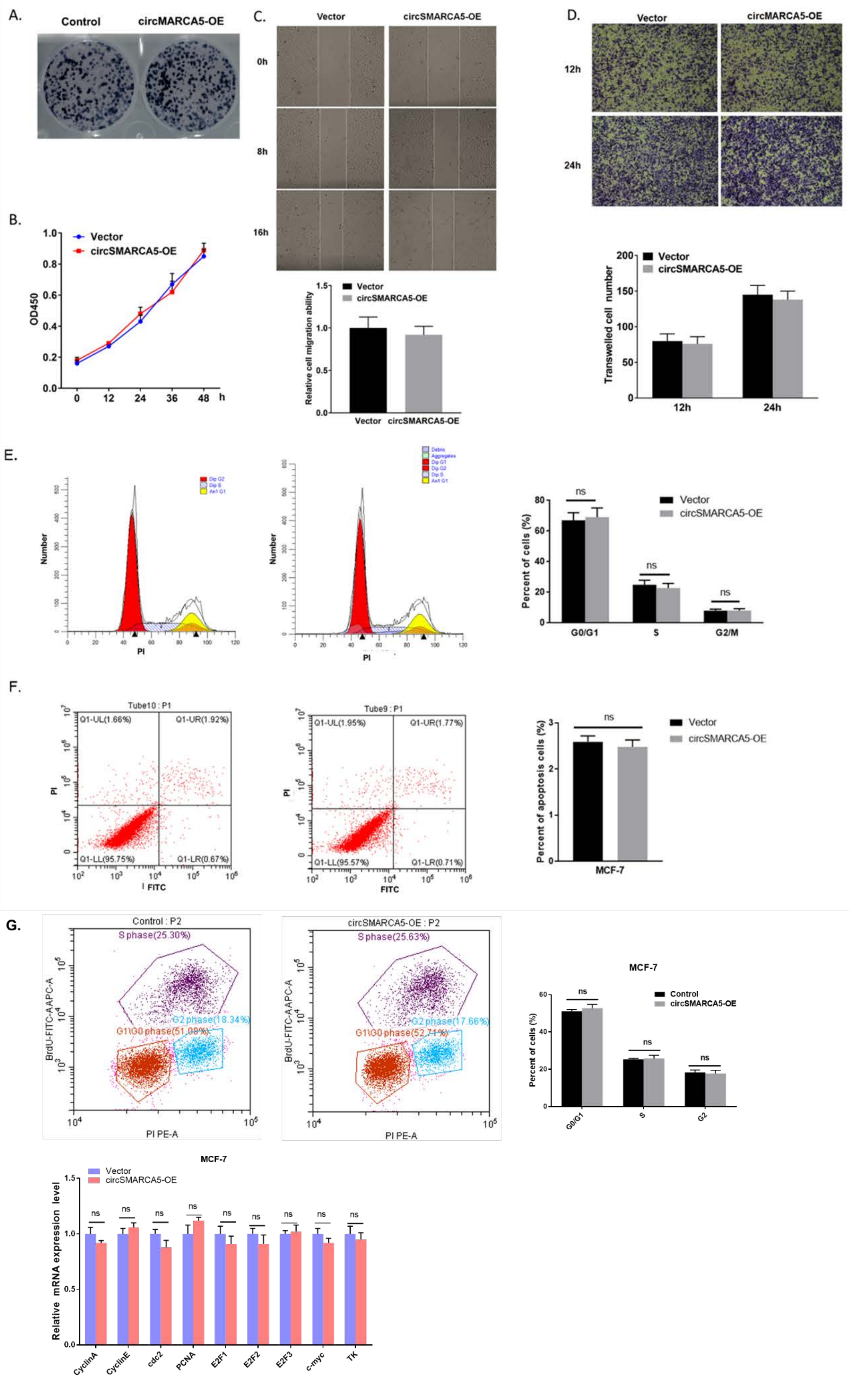

A.
Control
circMARCA5-OE
B.
Vector
circSMARCA5-OE
OD450
h
C.
Vector
circSMARCA5-OE
0h
8h
16h
Relative cell migration ability
D.
Vector
circMARCA5-OE
12h
24h
Transwelled cell number
E.
Number
PI
Percent of cells (%)
ns
G0/G1
S
G2/M
F.
Tube10 : P1
Q1-UL(1.66%)
Q1-UR(1.92%)
Q1-LL(95.75%)
Q1-LR(0.67%)
Tube9 : P1
Q1-UL(1.95%)
Q1-UR(1.77%)
Q1-LL(95.57%)
Q1-LR(0.71%)
PI
FITC
Percent of apoptosis cells (%)
MCF-7
G.
Control : P2
circSMARCA5-OE : P2
S phase(25.30%)
G2 phase(18.34%)
G1\G0 phase(51.08%)
S phase(25.63%)
G2 phase(17.66%)
G1\G0 phase(52.71%)
BrdU-FITC-AAPC-A
PI PE-A
MCF-7
Control
circSMARCA5-OE
Percent of cells (%)
G0/G1
S
G2
MCF-7
Vector
circSMARCA5-OE
Relative mRNA expression level
CyclinA
CyclinE
cdc2
PCNA
E2F1
E2F2
E2F3
c-myc
TK

Figure S9. circSMARCA5 has no significant effect on the proliferation and migration ability of breast cancer cells. (**A**) Relative colony formation units of control (pLCDH-ciR) or circSMARCA5 (pLCDH-circSMARCA5)-transfected stable MCF-7 cells. (**B**) The cell viability of MCF-7 cells expressing vector control (pLCDH-ciR) or circSMARCA5 (pLCDH-circSMARCA5) was determined by CCK8 assays at the indicated time points. (**C**) MCF-7 cells expressing vector control (pLCDH-ciR) or circSMARCA5 (pLCDH-circSMARCA5) were analyzed by wound healing assay. The percentage of wound closure was measured in triplicate experiments. (**D**) MCF-7 cells expressing vector control (pLCDH-ciR) or circSMARCA5 (pLCDH-circSMARCA5) were subjected to a migration assay. For the upper, representative photographs were taken at x200 magnification. The number of migrated and invaded cells was quantified in 4 random images from each group. (**E**)MCF-7 cells expressing vector control (pLCDH-ciR-GFPmut, without GFP fluorescence) or circSMARCA5 (pLCDH-circSMARCA5-GFPmut, without GFP fluorescence), cell cycle was detected by flow cytometry, each group was repeated for three times. (**F**)MCF-7 cells expressing vector control (pLCDH-ciR-GFPmut) or circSMARCA5 (pLCDH-circSMARCA5-GFPmut), cell apoptosis was detected by flow cytometry, each group was repeated for three times. **(G)** MCF-7 cells were transfected with control vector or circSMARCA5 overexpression vector. Cell cycle was analyzed by flow cytometry with BrdU-APC and PI stained, each group was repeated for three times. Cell cycle related genes are detected by RT-qPCR. “ns”: *P* >0.05, not statistically significant.

Figure S10

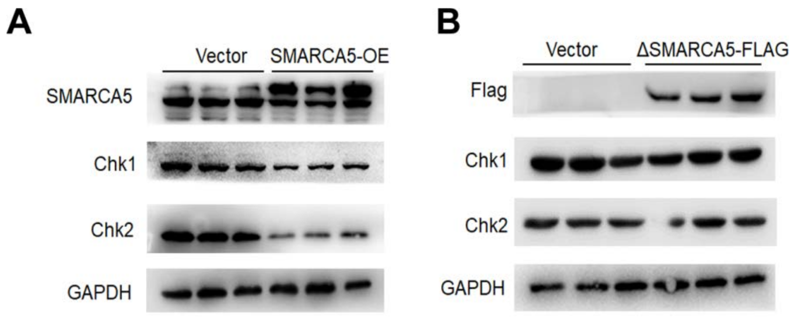

Figure S10.The truncated protein ΔSMARCA5 is a nonfunctional protein product. (**A**, **B**) The effect of SMARCA5 or ΔSMARCA5 overexpression on the expression of Chk1 and Chk2 after DNA damage repair. MCF-7 cells stably expressing Flag-SMARCA5 or Flag-ΔSMARCA5 were treated with 20 μM cisplatin for 24 h and replaced by fresh medium. After 24 h, the DNA damage markers Chk1 and Chk2 were detected by westernblot.

Figure S11

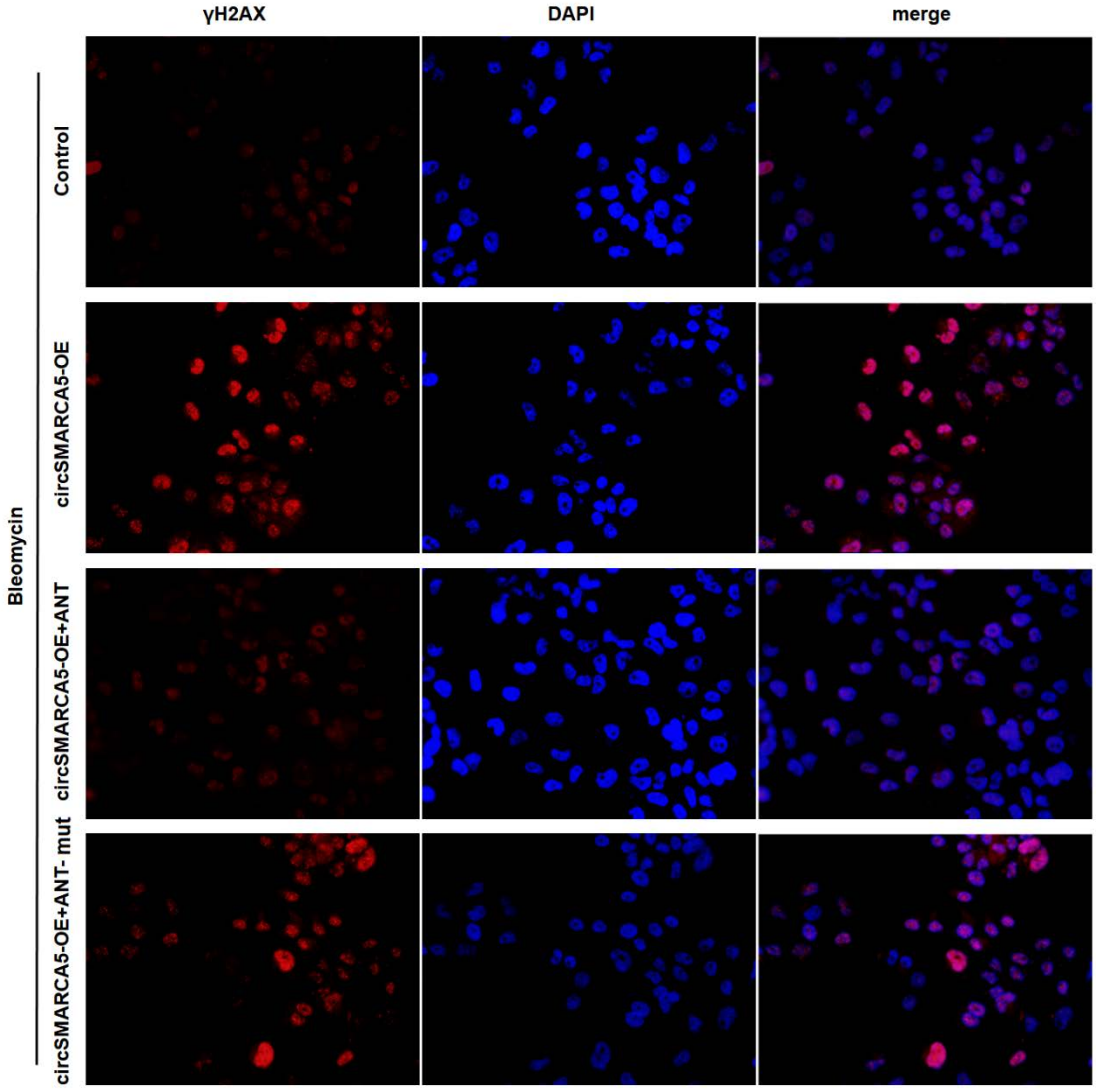

Figure S11. Immunofluorescence assay using a γH2AX antibody showing that the cotransfection of ANT in circSMARCA5-overexpressing cells can abrogate γH2AX levels inducedby circSMARCA5 but the cotransfection of ANT-mut cannot.

Figure S12

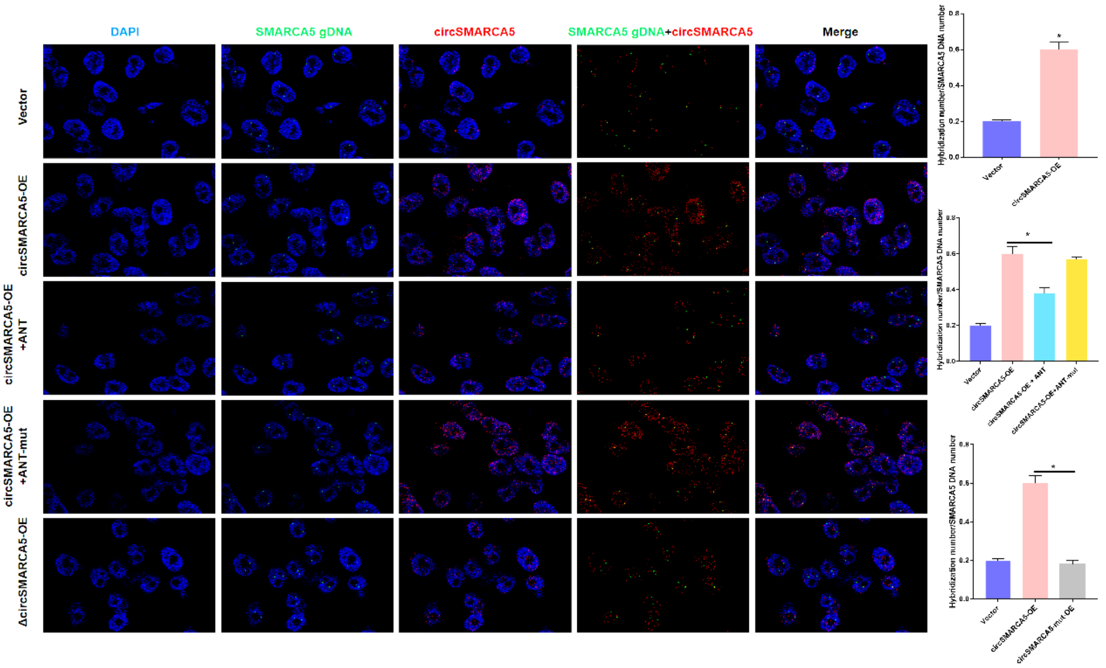

Figure S12. ANT significantly decreased the degree of colocalization between circSMARCA5 and its cognate DNA locus. MCF-7 cells were transfected with different combinations of molecules as indicated. Double FISH assay showing the localization of circSMARCA5 (red) and its cognate DNA locus (green). The nucleus was counterstained with DAPI. The histograms on the right panel are statistics of the number of sites colocalized circSMARCA5 and SMARCA5 genomic DNA.

Figure S13

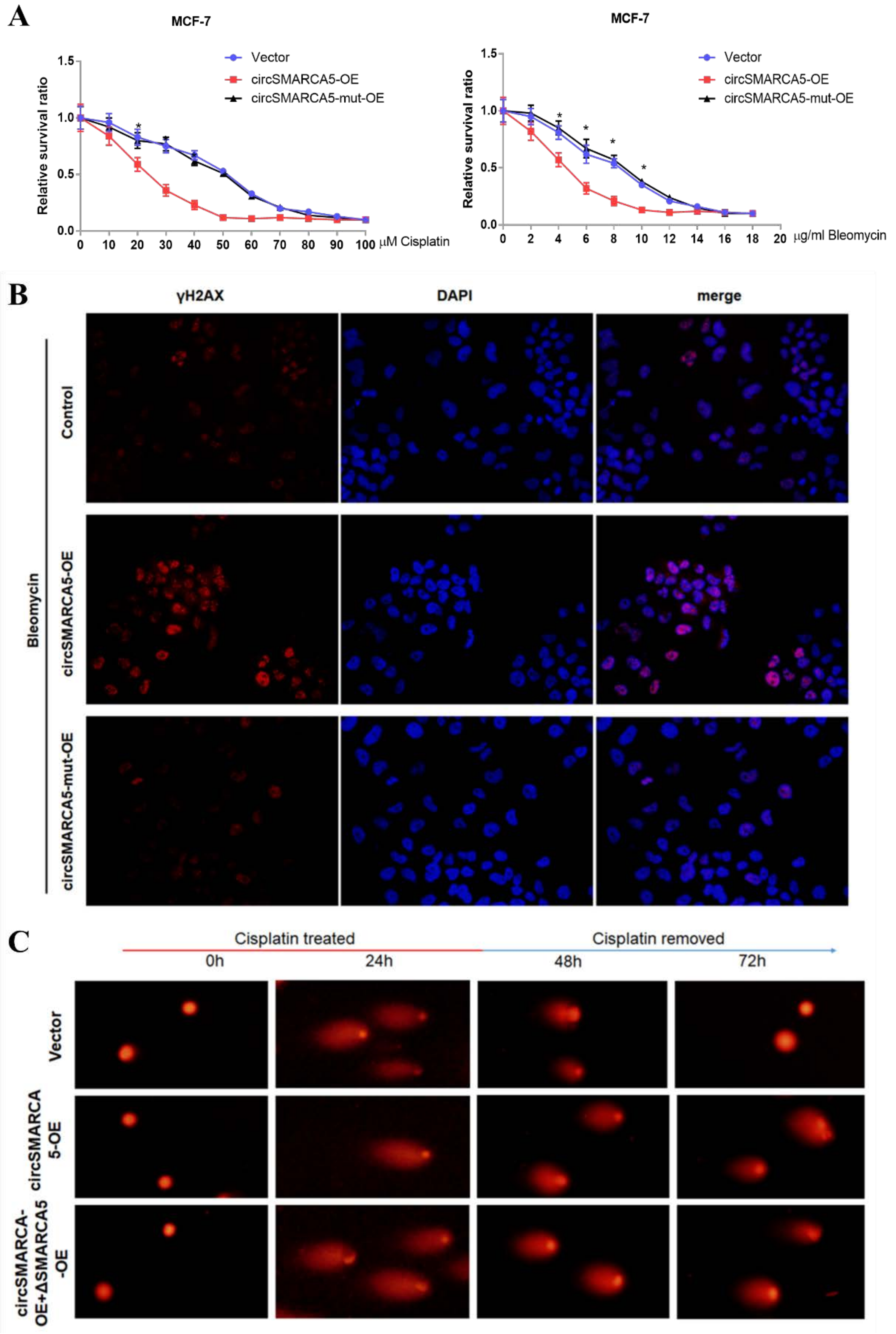
A
MCF-7
Vector
circSMARCA5-OE
circSMARCA5-mut-OE
Relative survival ratio
μM Cisplatin
MCF-7
μg/ml Bleomycin
B
γH2AX
DAPI
merge
Control
circSMARCA5-OE
circSMARCA5-mut-OE
Bleomycin
C
Cisplatin treated
Cisplatin removed
0h
24h
48h
72h
Vector
circSMARCA 5-OE
circSMARCA-OE+ΔSMARCA5 -OE

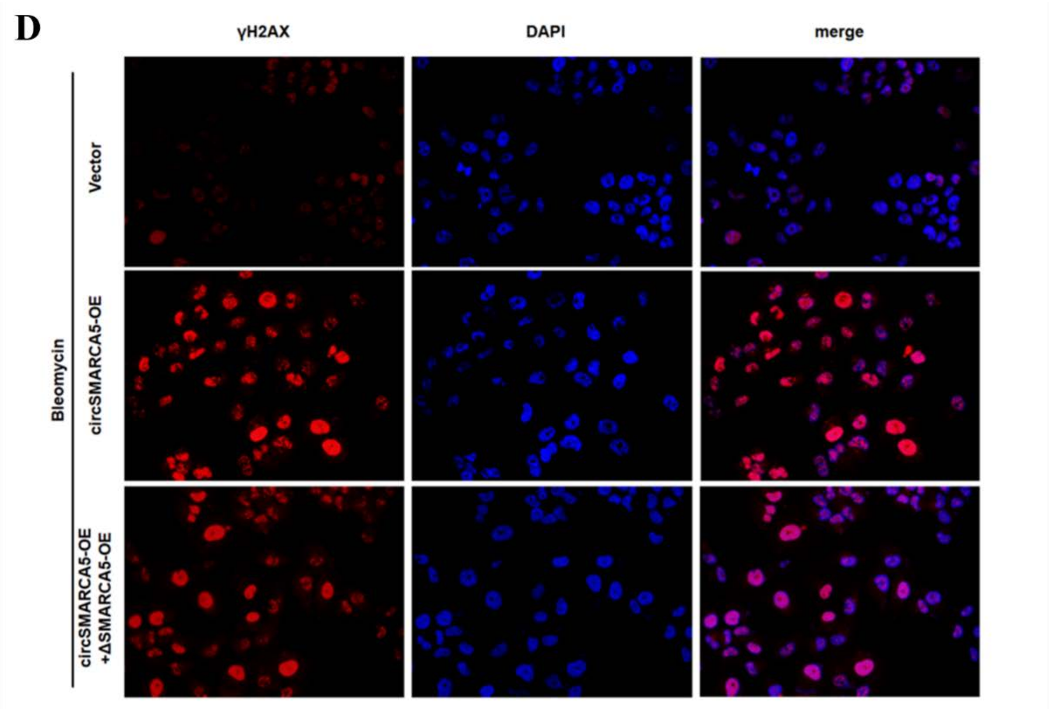

Figure S13. (**A**) MCF-7 cells expressing control vector, circSMARCA5 or circSMARCA5-mut were treated with cisplatin or bleomycin in concentration gradient for 24 h, and CCK8 was used to measure cell viability. (**B**) MCF-7 cells expressing control vector, circSMARCA5 or circSMARCA5-mut were treated with 6 μg/ml bleomycin. After incubation for 24 h, the cells were recovered with fresh medium for 48 hours and then collected for immunofluorescence assay using a γH2A antibody. **(C)** ΔSMARCA5 cannot restore the DNA repair capacity inhibited by circSMARCA5. MCF-7 cells were infected with different combinations of lentivirus as indicated and treated with 20 μM cisplatin. After incubation for 24 h, the cells were recovered with fresh medium for 48 hours and then collected for SCGE experiments. **(D)** ΔSMARCA5 cannot abrogate γH2AX levels induced by circSMARCA5. MCF-7 cells were infected with different combinations of lentivirus as indicated and treated with 6 μg/ml bleomycin. After incubation for 24 h, the cells were recovered with fresh medium for 48 hours and then collected for immunofluorescence assay using ananti-γH2AX antibody.

Figure S14

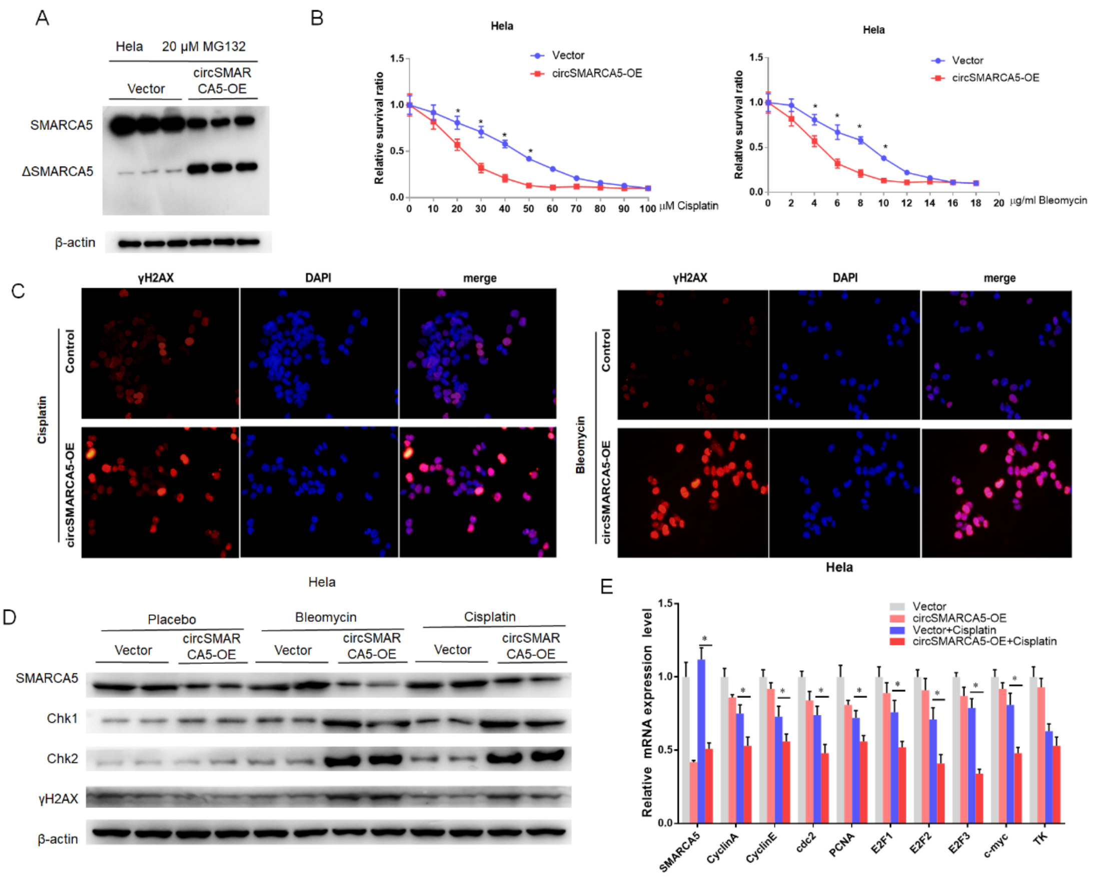

Figure S14. circSMARCA5 downregulate SMARCA5 and suppress DNA damage repair in Hela cell. (A) The full-length protein and defective protein were detected by western-blot after the Hela cells were treated by 20μM MAG132. ΔSMARCA5 represent the defective protein. (B) circSMARCA5 increases sensitivity to cisplatin or bleomycin in Hela. Hela cells stably expressing control vector or pLCDH-circSMARCA5 were treated with cisplatin or bleomycin for 24 h, and CCK8 was used to measure cell viability. (C) Relative colony formation units of MCF-7 cells stably expressing control vector or pLCDH-circSMARCA5 treated with 20 μM cisplatin or 6 μg/ml bleomycin. After 24 hours, the drugs were replaced by fresh medium. The immunofluorescence assay using an anti-γH2AX antibody (D)Western blot assay and RT-PCR with the indicated antibodies or primers of DNA damage repair and cell cycle pathway. *: $P < 0.05$ was considered statistically significant.
