# Supplementary material for: CircRNA inhibits DNA damage repair by interacting with host gene
Source: Mol Cancer. 2020 Aug 24;19:128. doi: 10.1186/s12943-020-01246-x (PMC7446195; doi:10.1186/s12943-020-01246-x)
Supplement: Supplementary file 2 — Additional file 2: Table S1. The ratio of circ-to-linear of circSMARCA5 (expression of circRNA / linear host genes) and clinical pathologic characteristics of breast cancer. [file 12943_2020_1246_MOESM2_ESM.docx]

Table S1．The ratio of circ-to-linear of circSMARCA5 (expression of circRNA / linear host genes) and clinical pathologic characteristics of breast cancer.

|  | **Various** | **Number** | **ratio of circ-to-linear** | | **P value** |
| --- | --- | --- | --- | --- | --- |
|  |  |  | **Low** | **High** |  |
| **Age** | | | | | |
|  | <60 | 10 | 6 | 4 | 0.679 |
|  | ≥60 | 14 | 6 | 8 |  |
| **Pathologic M** | | | | | |
|  | M0 | 16 | 8 | 8 | 0.665 |
|  | M1 | 8 | 4 | 4 |  |
| **Pathologic N** | | | | | |
|  | N0/N1 | 15 | 6 | 9 | 0.399 |
|  | N2/N3 | 9 | 6 | 3 |  |
| **Pathologic T** | | | | | |
|  | T1 | 14 | 4 | 10 | 0.038 |
|  | T2/T3/T4 | 10 | 8 | 2 |  |
| **ER status** | | | | | |
|  | Negtive | 9 | 6 | 3 | 0.399 |
|  | Positive | 15 | 6 | 9 |  |

Pathologic M: Evaluation by remote metastasis; Pathologic N: Evaluation by lymph node infiltration and metastasis; Pathologic T: Evaluation by tumor volume and tumor infiltration range; ER status: Evaluation by immunohistochemistry of estrogen receptor; *P* values were based on χ2-test, *P* < 0.05 was considered statistically significant.
